# Phytohormone Priming of Tomato Plants Evoke Differential Behavior in *Rhizoctonia solani* During Infection, With Salicylate Priming Imparting Greater Tolerance Than Jasmonate

**DOI:** 10.3389/fpls.2021.766095

**Published:** 2022-01-10

**Authors:** Paulami Koley, Subhadip Brahmachari, Amitava Saha, Camelia Deb, Monimala Mondal, Nebedita Das, Arpan Das, Suvanwita Lahiri, Mayukh Das, Manisha Thakur, Surekha Kundu

**Affiliations:** Molecular and Applied Mycology and Plant Pathology Laboratory, Department of Botany, University of Calcutta, Kolkata, India

**Keywords:** *Rhizoctonia*, tomato, defense, salicylic acid, jasmonic acid, priming, necrotroph, hemibiotroph

## Abstract

In the field of phytohormone defense, the general perception is that salicylate (SA)-mediated defense is induced against biotrophic pathogens while jasmonate (JA)-mediated defense functions against necrotrophic pathogens. Our goals were to observe the behavior of the necrotrophic pathogen *Rhizoctonia solani* in the vicinity, on the surface, and within the host tissue after priming the host with SA or JA, and to see if priming with these phytohormones would affect the host defense differently upon infection. It was observed for the first time, that *R. solani* could not only distinguish between JA versus SA-primed tomato plants from a distance, but surprisingly avoided SA-primed plants more than JA-primed plants. To corroborate these findings, early infection events were monitored and compared through microscopy, Scanning Electron Microscopy, and Confocal Laser Scanning Microscopy using transformed *R. solani* expressing green fluorescence protein gene (gfp). Different histochemical and physiological parameters were compared between the unprimed control, JA-primed, and SA-primed plants after infection. The expression of a total of fifteen genes, including the appressoria-related gene of the pathogen and twelve marker genes functioning in the SA and JA signaling pathways, were monitored over a time course during early infection stages. *R. solani* being traditionally designated as a necrotroph, the major unexpected observations were that Salicylate priming offered better tolerance than Jasmonate priming and that it was mediated through the activation of SA-mediated defense during the initial phase of infection, followed by JA-mediated defense in the later phase. Hence, the present scenario of biphasic SA-JA defense cascades during *R. solani* infection, with SA priming imparting maximum tolerance, indicate a possible hemibiotrophic pathosystem that needs to be investigated further.

## Introduction

After the initial recognition of the pathogen on the cell surface, plants deploy their second phase of defense through the activation of a complex network of signaling cascades directed by phytohormones. The two major players of this phytohormone-mediated plant immunity are salicylic acid (SA) and jasmonic acid (JA) (Yang et al., 2019a). It has been suggested that SA and JA are mutually inhibitory for the expression of many genes (Glazebrook, 2005) and, hence, are mutually antagonistic in function. Therefore, plants must deploy this repertoire of signaling cascades in such a way as to minimize its cost with maximum benefit (Huang et al., 2020).

The general perception is that effective defense against biotrophic pathogens is largely attributed to localized programmed cell death in the host followed by activation of defense cascade mediated through SA. In contrast, host cell death is advantageous for necrotrophic pathogens. Therefore, they are thwarted by a different set of defense responses activated by JA and ethylene signaling (Glazebrook, 2005). The hemibiotrophic pathogens, on the other hand, deploy a coordinated strategy of infection where an initial biotrophic mode is followed by necrotrophic mode (Hane et al., 2020; Précigout et al., 2020; Jones et al., 2021), separated by a short biotrophy-necrotrophy switch (Chowdhury et al., 2017a). Accordingly, the host also utilizes both SA and JA-mediated defense signaling pathways in a sophisticated and coordinated manner to counter these pathogens during different stages of infection (Ding et al., 2011; Mencia et al., 2020).

The JA- and SA-mediated defense signaling is routed through complex pathways involving key marker genes. The presence of a necrotroph on the host surface induces host systemin receptors like SR160 and is recognized by the CORONATIN INSENSITIVE 1 (COI1) receptor in a jasmonate-dependent manner. COI1-JA complex is first formed, which subsequently recruits JAZ repressors for further signal transduction (Yan et al., 2018). COI1 then directs the ubiquitin mediated proteosomal degradation of JAZ repressors to activate the downstream expression of jasmonate responsive genes (Antico et al., 2012; Li et al., 2019; Yang et al., 2019a). Concurrent upregulation of COI1 and JAZ in response to JA treatment and stress have been reported (Zhang et al., 2012). Therefore, a complex regulation exists for these components and information about the interaction mode of this complex is still scarce at the molecular level (Garrido-Bigotes et al., 2020). The primary signal involving these three components is further routed in two directions with one leading to the enhanced production of endogenous JA in the chloroplast through the JA synthesizing enzymes like *AOS* (Han, 2017; Ruan et al., 2019). The other route is the transcription of defense marker genes viz. *PDF1.2* (Proietti et al., 2018) and *PINII* (Rehman et al., 2017).

Methyl jasmonate (MeJA) is a common derivative of endogenous JA that acts as a functional analog which, when applied exogenously, can successfully induced this entire JA-mediated response against necrotrophic pathogens like *Botrytis cinerea* (Hu et al., 2018; Yu et al., 2018), *Sclerotinia sclerotiorum* (Oliveira et al., 2015), *Alternaria brassicicola* (Brenya et al., 2020), and *Rhizoctonia solani* (Kidd et al., 2021). Contrastingly, SA-mediated signaling pathways have long been designated for the development of systemic acquired resistance (SAR) in response to biotrophic pathogens accompanied by the concomitant induction of pathogenesis-related (PR) protein genes, especially *PR1* (Ali et al., 2018; Betsuyaku et al., 2018; Zhang et al., 2019). Important genes from SA synthesizing pathways, like Isochorismate Synthase (*ICS*) and Phenylalanine Ammonia-Lyase 5 (*PAL5*), along with downstream SA responsive marker genes Like Phytoalexin-Deficient 4 (*PAD4*), have long been known to be induced substantially in response to biotrophs (Cui et al., 2017; van Butselaar and van den Ackerveken, 2020).

Priming of plants with pytohormones to induce their defense response, followed by infection with pathogen, can be an efficient method when studying the host defense responses in detail. However, most of the studies to date in this field were focused on increasing resistance of hosts toward their pathogen through phytohormones priming (Conrath et al., 2015; Bawa et al., 2019). Previous studies have established that application of MeJA conferred resistance against necrotrophic pathogen, while that of SA conferred resistance against biotrophic pathogen (Oliveira et al., 2015; Chen et al., 2017; Yang et al., 2019b).

In spite of many studies on necrotrophic diseases, there are only a handful of studies where pathogen behavior was monitored during the early stages of infection. In our previous reports, we have shown that there was a significant difference in the behavior of the pathogen when it interacted with or was in the vicinity of a resistant versus a susceptible host (Chowdhury et al., 2014; Ray et al., 2015; Basu et al., 2016; Chowdhury et al., 2017a). To date, there is no study on the behavior of the pathogen in the vicinity, on the surface, and within the tissue of the host plant that has been primed with phytohormones. Therefore, with our previously standardized methods, our aim was to see for the first time whether the pathogen can distinguish between a primed host versus an unprimed control host and behave differently. Moreover, our plan was to also prime the host separately with two different phytohormones, JA and SA, followed by infection with the necrotrophic pathogen *Rhizoctonia solani* and then study the subsequent host defense signaling. We wanted to see how SA priming would affect the defense against a necrotroph. Most importantly, we were also interested in comparing the magnitude of defense provoked against a necrotrophic pathogen after priming with JA with that provoked after priming with SA, which has not been studied thus far.

## Materials and Methods

### Growth Conditions for Tomato Plants and the Pathogen *R. solani*

Tomato plants of Pusa Ruby variety (PR) (Sutton Pvt. Ltd., India) was grown on commercial soilrite (a mixture of horticulture grade perlite, Irish peat moss and exfoliated vermiculite in 1:1:1 ratio) and maintained in plant growth chambers with 16 h light and 8 h dark cycles, at a temperature of 26 ± 1°C under a light intensity of 50 μM m^–2^ s^–1^. Pure culture of the pathogen *Rhizoctonia solani* Kuhn. (AG1-1A isolate of the fungus was obtained from Rice Research Station, Chinsurah, West Bengal, India, Basu et al., 2016) was used for this study. The fungus was maintained at 28°C in potato dextrose agar plates. For inoculation of plants, both sclerotia and mycelial discs of 3 mm diameter from the growing edge of 10-day-old culture were used according to our previous report (Basu et al., 2016).

### Phytohormone Priming of Tomato Plants

Preparation of hormone solutions was done as per our earlier published article (Chowdhury et al., 2017a). Tomato plants were primed with either of the two phytohormones, MeJA (Sigma-Aldrich) or SA (Himedia, India). For priming of plants, 0.08 mM concentration of the phytohormones was used as it did not affect the fungal growth when the media was supplemented with the phytohormones at this concentration (Supplementary Figures 1–3). One hundred millimolars of phytohormone stocks were prepared by dissolving in ethanol. This stock was used to make the final concentrations in the experiment in distilled water. Hormone solutions were sprayed according to Chowdhury et al., 2017a on 4-week-old plants and covered for 24 h prior to inoculation with *R. solani.* Control plants of similar age were sprayed with the diluted solvent only, without the phytohormones.

### Assay of Pathogen Behavior in the Vicinity and on the Host Surface

This study was carried out using detached leaf assay according to our earlier published protocol (Basu et al., 2016). For behavior of pathogen in the vicinity of leaves, fully expanded leaves from 4-week-old plants were placed on water agar media in petri plates, keeping the dorsal sides up. One 3 mm mycelial disc was placed 1 cm away from the edge of the leaf and allowed to grow toward the leaf. Three microscopic fields were observed for each leaf at 3 and 5 dpi. Photographs were taken with the help of a stereomicroscope (Radical Ltd., India). Results were obtained from three independent experiments three replicates each.

### Evaluation of Sclerotial Germination Over a 24-h Time Course Post Inoculation

This was done according to our protocol (Basu et al., 2016). Sclerotia of similar sizes were placed singly at the center of tomato leaves of control, MeJA, and SA-primed leaves. Leaves were incubated at 26°C (± 1°C) in petri plates with humidity maintained by moist tissue papers. Germination of these sclerotia and progression of the emerging hyphae was observed at 4-h intervals (4, 8, 12 and 24 hpi) by stereomicroscope (Radical Ltd., India) and compound microscope (Leica DMLS). For each priming regime, three independent experiments with three replicates each were used for data analysis.

### Preparation of Samples for Scanning Electron Microscopy

Leaves were prepared for scanning electron microscopy (SEM) according to Basu et al., 2016. First, the leaves were fixed using 3% glutaraldehyde in0.1 M sodium cacodylate buffer (pH 7.2) overnight at 4°C. The next day, the excess fixative was removed by washing the leaves thoroughly with0.1 M sodium cacodylate. Then, the leaves were dehydrated with a serial dilution in an ethanol series of 25, 50, 70, 85, 95, and 100% v/v. The plant samples were processed through a critical point drying technique and then mounted on metal stubs coated with gold. Samples were observed under a scanning electron microscope (Carl Zeiss EVO 18, Germany).

### Confocal Laser Scanning Microscopy of Tomato Leaves and Stems Infected With *R. solani* Transformed With Green Fluorescence Protein Gene

For confocal microscopy, tomato plants were inoculated with mycelial discs of *R. solani* transformed with *gfp* gene. *R. solani* had been previously transformed in our laboratory by Basu et al., 2016. Infected leaf and stem samples were processed for removal of chlorophyll through acetoethanol (1:3, v/v). All the samples were subjected to CLSM analysis using the model IX81, Olympus Singapore Pte Ltd., equipped with FLUOVIEW FV1000 software. Representative fluorescence fields were chosen from at least three independent plants. For visualization of green fluorescence protein (GFP) fluorescence, the excitation wavelength was 473 nm and the emission window was set at 485–585 nm.

### Calculation of Disease Index

Disease index was calculated according to Basu et al., 2016. The characteristic brown colored lesions that developed on tomato leaves during *R. solani* infection was considered for disease index study on both detached leaves and whole seedlings. Nine plants were observed, and the experiment was repeated four times. Six-week-old seedlings were inoculated with single sclerotium at the center of each leaf to calculate disease index of whole seedling. For detached leaf assay, each leaf was inoculated with a sclerotium at the center. After inoculation, the whole seedlings and detached leaves were incubated at 26 ± 1°C in humid condition. The area of the brown necrotic lesions developed on seedlings and detached leaves were calculated and represented as the percentage of necrotic area against the whole leaf area. Disease index necrotic areas of the infected leaves at 2 or 3 dpi was graded into five classes (0 = no infection, 1 = 1–25%, 2 = 26–50%, 3 = 51–75%, and 4 = 76–100% infected leaf area). Disease index (DI) was calculated according to Basu et al., 2016.

### Quantitative Evaluation of Formation of Infection Cushions

Tomato leaves of control and experimental sets were collected at different time points post-inoculation (12, 24, 48, and 72 hpi). After removal of chlorophyll with acetoethanol solution (1:3), leaves were stained with lactophenol-trypan blue solution (0.5% trypan blue, lactic acid:phenol:H_2_O 1:1:1 v/v/v) and kept overnight. The excess stain was later washed off with distilled water and the leaves were mounted with 50% glycerol on glass slides for viewing under compound microscope (Olympus BX-51). Number of infection cushions per microscopic field on the leaf surfaces was counted. Each leaf was scored for three microscopic fields. Three leaves taken from three different experimental sets each of control and primed plants were evaluated for each time point. Evaluation was done according to Basu et al., 2016.

### Detection of Polyphenol and Peroxide Accumulation and Callose Deposition in Leaves

Cellular deposition of phenolics, peroxide, and callose was detected by toluidine blue and DAB stain according to our protocol (Ray et al., 2015), and aniline blue (Chowdhury et al., 2017a) respectively. Six independent experiments with two leaves each were used for polyphenol and peroxide accumulation study. For both assays, chlorophyll from leaves was removed with 1:3 aceto-ethanol solution. For detection of polyphenol, leaves were immersed in 0.05% toluidine blue stain (dissolved in 0.1 M potassium phosphate buffer, pH 5.5) for 6 hours. Accumulation of phenols was indicated by blue patches formed on the leaf surface.

Peroxide was detected by soaking the leaves in 1 mg/ml 3’3-Diaminobenzidine (DAB) solution (pH 3.8) for 8 h. The dark brown patches on the leaf surface indicated the accumulation of peroxide. Data were presented as the percentage of colored area with respect to total leaf area.

Pathogen-mediated callose deposition on the leaves was observed under a fluorescence microscope (Leica DMLS, excitation maximum 330–385nm, dichroic mirror DM 400, barrier filter > 420 nm). Deposited callose in the leaves was observed for the bluish-green fluorescence developed after aniline blue staining. Four leaves were observed with three microscopic fields from each leaf according to Chowdhury et al., 2017a and representative fields were considered.

### Determination of Total Phenol, Flavonoid, Proline, Malondialdehyde Content (Lipid Peroxidation), and Chlorophyll Content

These assays were done according to our standardized protocols (Ray et al., 2015; Chowdhury et al., 2017a,b) with three independent experiments using at least three individual plants in each experiment. For quantification of total phenol, leaves were homogenized in 80% methanol. Tissue extracts were then mixed with water and folin-ciocalteau (Sigma Aldrich) reagent followed by 5 min of dark incubation. The reaction was started after the addition of 20% sodium-tartarate followed by 30 min of incubation at room temperature. Absorbance was recorded at 720 nm, and the amount of total phenol was expressed per gram of fresh tissue against the standard curve of gallic acid.

Flavonoid was quantified according to our protocol (Chowdhury et al., 2017a,b), by extracting plant tissue in 80% ethanol. A reaction mix was made containing 500 μl tissue extract along with 10% aluminum chloride, 1 mM potassium acetate, and distilled water to a final volume of 5 ml. Data was collected as the absorbance at 415 nm and represented per gram of fresh tissue according to our Chowdhury et al. (2017a,b).

Proline and malondialdehyde contents were assayed in accordance with Chowdhury et al. (2017b).

Total chlorophyll content was determined by extraction of fresh leaves in 80% acetone according to Chowdhury et al. (2017b).

### Quantitative Assay of Expression of Defense Genes *via* Real Time PCR

This was done according to our published protocol (Chowdhury et al., 2017a,b). Total RNA was isolated from leaf samples by Trizol (Invitrogen) method, followed by quantification with a nanodrop spectrophotometer (Eppendorf). Two micrograms of RNA were used to synthesize cDNA using random primers. Real time PCR (RT-qPCR) was carried out in 96-well blocks in 20μl reaction volume (Applied Biosystems 7500 Real-Time PCR System, USA) using Power SYBR Green master mix (Applied Biosystems, USA). Transcript sequences were amplified by gene-specific primers. Relative fold changes in gene expression were determined by a standard method, particularly by calculating ΔΔCt value. The specificity of the PCR reactions was adjusted based on a melting curve analysis of the amplified products utilizing the standard method installed in the system. The pathogen gene 18s rDNA was used as an internal control for the pathogen (Sayler and Yang, 2007). Tomato actin gene was used as internal control according to our protocol (Ray et al., 2015). The list of primers both for the pathogen and the tomato plants used in this study is given below.

List of primers used for quantitative real-time PCR:

**Table:** 

No	Name of Genes	Forward (5′-3′)	Reverse (5′-3′)
**Primer used for *Rhizoctonia solani***			
1	*RsGAS1*	GCCGTTCTCGCC CTTGA	TCGCAGGTCGCT TCCATT
2	*RsAG1IA rDNA*	GCCTTTTCTACCTTAATT TGGCAG	GTGTGTAAATTAAGTAGAC AGCAAATG
**Primer used for tomato plants**			
3	*SlCOI1*	TCGGCATTGTTGTTG TTGTT	TTGAGAGGTAGAGG GGCTGA
4	*SlAOS*	GAAGTCGGTGCATCT CCATT	CGGCATGCTCTGTTC TGTAA
5	*SlJAZ2*	CGTCCGTTGAAACA AATCCT	GGGGTTCTGTTTGT TGGCTA
6	*SlPIN II*	TGTTGATGCCAAGG CTTGTA	CTTGTGAACGGG GACATCTT
7	*SlSR160*	TGGCCATGGGTTTG TTATTT	CTCAAATGCTGCA AGGTTGA
8	*SlCHS1*	CAGTACTTCGGCTAGCC AAGGA	CGGAACGTAACT GCAGTGATCT
9	*SlCHS2*	GGCCTTTGTTTGAACTC GTCTCT	GCGAAGGTGACC ATCAATAGC
10	*SlBSMT*	ATCAGGCGTTCCAGG TACCTT	AGGAGCCTGAGAT AGCCAATGA
11	*SlICS*	TCATTAGACGATTGGC GTGCTA	GCTGTTGCATCAA ATCGGATT
12	*SlPAD4*	AACATTTGGCTCTCC AATGC	CTCCCAGAGGGACA AAATGA
13	*SlPAL5*	AACAGCAACATTACCC CGTGTT	GCAATGTATGACAACG GGACAA
14	*SlPR1a*	TGGTATGGCGTAAGT CGGTA	CTTGGAATCAAAGT CCGGTT
15	*SlActin*	GGATCTTGCTGGTC GTGATT	CTTGTCCATCAG GCAATTCA

### Assay of Effect of Methyl Jasmonate and Salicylic Acid on the Growth of *R. solani*

The effect of MeJA and SA on the growth of *R. solani* was determined by allowing the fungus to grow on potato dextrose agar (PDA) plates supplemented with varying concentrations of MeJA or SA. The concentrations used were 0.05, 0.08, 0.1, 0.5, and 1 mM for each of the phytohormones. A mycelial disc of 3 mm diameter from the growing culture of *R. solani* was placed in the center of each phytohormone supplemented PDA plate. These plates were incubated in 28°C for the next three days and colony diameter was recorded. Data were collected from three independent replicates.

### Quantification of Salicylic Acid and Jasmonate Content Under Different Experimental Conditions

Salicylic acid (SA) and JA were extracted from tomato leaves according to de Sa et al. (2013). For extraction of SA and JA, 500 mg tissue was ground in liquid nitrogen. Ground tissues were homogenized in 85% MeOH (v/v). Samples were then centrifuged at 10,000 rpm for 10 min at 4°C. The supernatants were mixed with0.2 mol/L NaOH. The mixtures were filtered using a Whatmann grade 1 filter paper. Filtrates were mixed with 5% Trichloroacetic acid (TCA) and were partitioned using ethyl acetate and cyclohexane (1:1 v/v). The organic phases were collected, and 0.1 mol/L sodium acetate was added and then dried. Dried samples were suspended in 10% methanol for analysis with HPLC. Three sets of extracted samples for each time point and SA and JA standards were analyzed with HPLC using standard calculations according to Trapp et al. (2014). HPLC system (Agilent Technologies, USA) with 4.6 × 100 mm, 3.5 μm column was used. Formic acid (0.05%, v/v) and MeOH with0.05% (v/v) of formic acid were used as mobile phases A and B, respectively. The elution gradient used was 0-10 min, 42-55% B in A; 0-13 min, 55-100% B; 13-15 min 100% B; 15-15.1 min 100-42% B in A; and 15.1-20 min 42% B in A at a constant flow rate of 1.1ml/min. All the chemicals were purchased from Sigma Aldrich and were of HPLC grade as required.

### Statistical Analysis

All the data were analyzed using software according to our previous publications (Chowdhury et al., 2017a,b). Each experiment was done in a completely randomized design (CRD) with three independent experiments with three to four replicates each and values represented as the standard error mean ± SEM. The data were subjected to one-way analysis of variance (ANOVA) with different letters indicating significant differences between treatments at *p* < 0.05, according to Duncan’s multiple range test (DMRT), using a software package, SPSS version 16, 2007.

## Results

### Differential Behavior of *R. solani* in the Vicinity of Phytohormone Primed and Control Host Leaves

**FIGURE 1:**
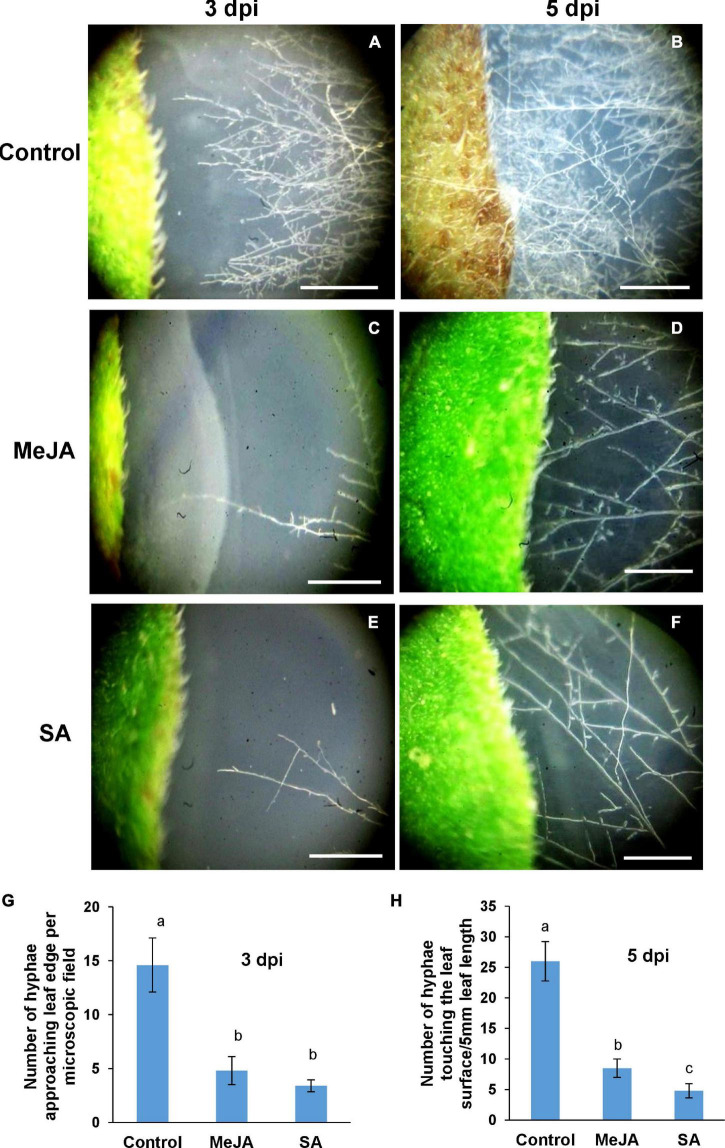
Differential behavior of *Rhizoctonia solani* hyphae in the vicinity of phytohormone primed and control leaves. **(A,B)** When allowed to approach the host leaves from a distance, *R. solani* hyphae showed more preference for the control leaves with more number of hyphae per unit area in the vicinity of the leaves. **(C,D)** The number of hyphae approaching and making contact with the edge of the leaf was significantly less in the case of the methyl jasmonate (MeJA)-primed leaves. **(E,F)** Least number of hyphae approached the salicylic acid (SA)-primed leaves where the hyphae grew parallel to the leaf edge, avoiding perpendicular contact. **(G)** Graph showing hyphal density in the vicinity of leaves at 3 dpi. **(H)** Graph showing number of hyphae making contact with the edge of the leaves at 5 dpi, with control showing maximum hyphae followed by MeJA-primed leaves and least in the SA-primed leaves. Bars represent mean ± S.E.M of three independent experiments with three replicates. Different letters indicate significant differences among treatments at *p* < 0.05 according to Duncan’s multiple range test. Bar = 2 mm.

We wanted to observe the behavior of *R. solani* and see if the fungus showed any preference toward unprimed versus primed host. The fungal hyphae were allowed to grow toward the host leaves from a distance (Figures 1A–F). We found that there was a marked difference in the behavior of *R. solani* in the vicinity of primed versus unprimed leaves which became more visibly pronounced at 3 and 5 dpi when the hyphae reached closer to the leaves (Figures 1A–F). In the case of control leaves, numerous hyphae approached the leaf, some of which almost touched the leaf edges by 3 dpi. In contrast, only a few hyphae reached the vicinity of MeJA and SA pretreated leaves (Figure 1G). Almost a 3 times greater number of hyphae grew in bulk toward the control leaves, straight from the germinating sclerotia approaching the leaf edge in a perpendicular direction by 5 dpi (Figure 1H). The SA-primed leaves were least preferred by the pathogen at 3 and 5 dpi (Figures 1G,H). In the case of the SA-treated leaves, the hyphae ran parallel to the leaf margin at a distance, avoiding direct contact even at 5dpi (Figure 1F). The infection progressed quickly in control leaves such that the leaves started to necrose on the 5th day of the experiment (Figure 1B). In congruence with the behavior of the pathogen in the vicinity, the phytohormone treated leaves remained green and fresh even at the end of the experiment at 5 dpi (Figures 1D,F).

### Behavior of Fungal Sclerotia on the Surface of Primed Host Leaves Compared to Control Leaves

**FIGURE 2:**
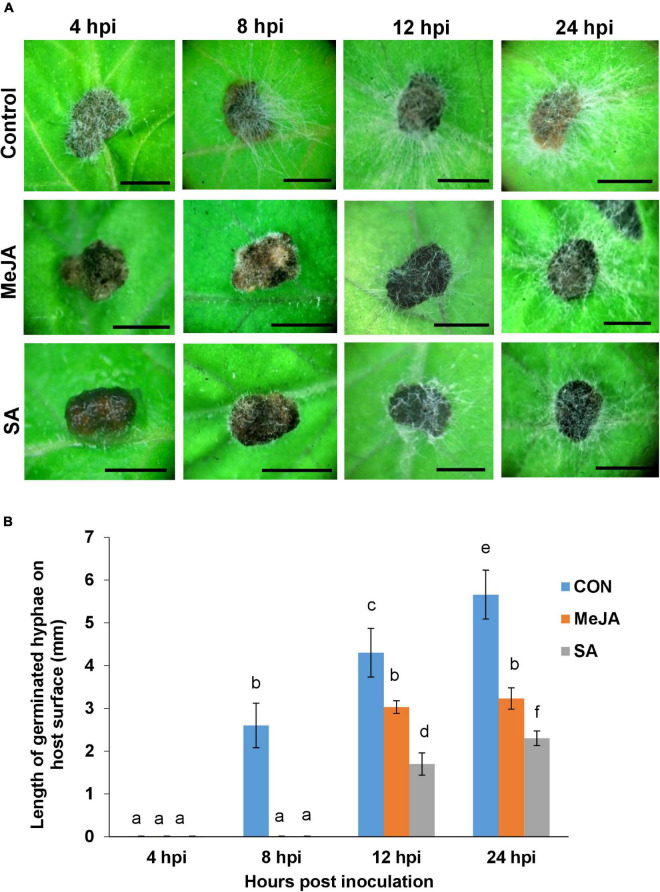
Behavior of germinating *R. solani* sclerotia on tomato leaves primed with MeJA or SA compared with the control observed over a 24 h time course. **(A)** Top row: Sclerotia starting to germinate vigorously within 4 h after placement on the control leaves. Profuse infection hyphae spreading in all directions almost covering the leaf surface by 12 and 24 hpi. Middle row: Sclerotia showing delayed germination on the MeJA-primed leaves with infection hyphae of measurable length being visible only from 12 hpi. Bottom row: Sclerotia showing the most delayed germination and the lowest amount of infection hyphae on the SA-primed leaves. **(B)** Graph showing the comparison of length of infection hyphae from germinating sclerotia on control, MeJA-primed, and SA-primed leaves. Bars represent mean ± SEM of three independent experiments with three replicates. Different letters indicate significant differences among treatments at *p* < 0.05 according to Duncan’s multiple range test. Bar = 1 mm.

For *R. solani*, the compacted mass of hyphae forming the sclerotia serve as primary inocula. In order to find out if host priming affected the behavior of the sclerotia, the steps of the sclerotial germination was studied on primed and unprimed control leaves. The lengths of hyphae from germinating sclerotia were measured at fixed time intervals in three experimental sets of control, MeJA, and SA-primed host leaves (Figure 2). In the control leaves, the sclerotia started to germinate as early as 4 h post-inoculation. The hyphae continued to spread out at 8 and 12 hpi. At 24 h, the sclerotia became obscured in the surrounding dense cottony mass of hyphae (Figure 2A). In contrast, the sclerotia did not germinate at 4 hpi and barely started to germinate at 8 hpi on the primed leaves. The emerging hyphae were visibly spreading onto the leaf surface only at 12 and 24 hpi, with much less growth compared to the control. The most surprising observation was that sclerotial germination was least in the SA pre-treated leaves compared to the MeJA pre-treated. There was nearly double the growth of hyphae from sclerotia placed on MeJA-primed leaves compared to SA-primed leaves. This shows that the MeJA pre-treatment was less of a deterrent compared to SA pre-treatment (Figure 2B).

### Scanning Electron Microscopy to Observe Differential Behavior of Fungal Sclerotia and the Newly Emerging Hyphae on Primed and Unprimed Control Leaves

**FIGURE 3:**
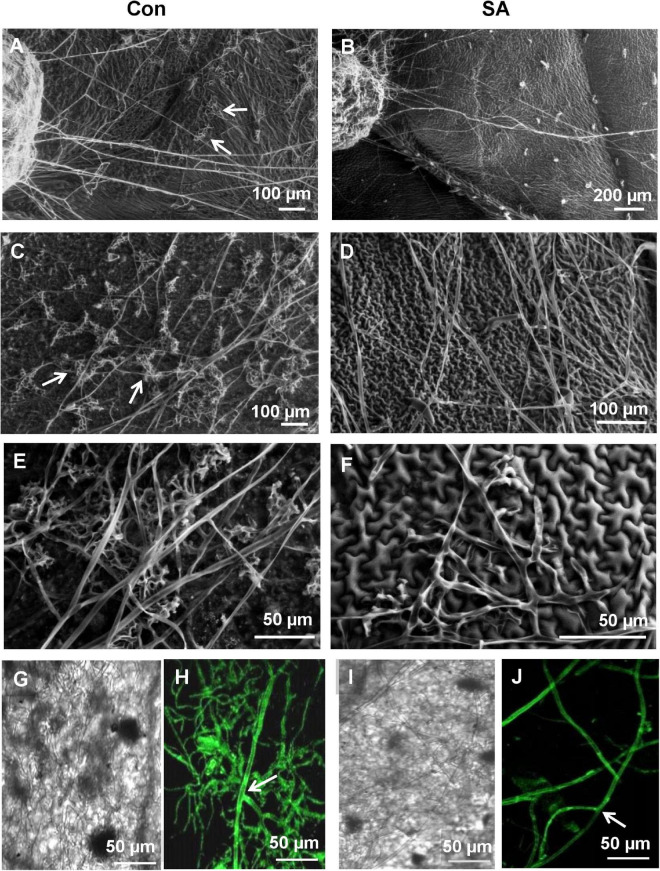
Scanning Electron Microscopy and Confocal Laser Scanning Microscopy (CLSM) to observe differential behavior of sclerotia and the newly emerging infection hyphae of *R. solani* transformed with *gfp* gene on SA-primed and control leaves up to 48 hpi. **(A)** SEM of germinating sclerotia showing profuse growth on the control leaf, presence of hyphal aggregation, and initiation of infection cushions (arrows) at 24 hpi. **(B)** Delayed germination of sclerotia with significantly less emerging hyphae and no infection cushion on the surface of SA-primed leaf at the same point **(C)** Numerous infection cushions on control leaf surface at 48 hpi **(D)** Hyphae growing in straight lines with scant hyphal branching and light hyphal aggregation at 48 hpi on SA pre-treated leaves. **(E)** SEM of close-up view of advanced infection cushion on the control leaf at 48 hpi **(F)** A close-up view of loose hyphal aggregation on SA-primed leaf at 48 hpi. **(G,H)** Confocal microscopy of *gfp*-transformed *R. solani* hyphae on control leaf surface at 24 hpi showing profuse branching, arrow showing right angled branching with septa typical for *R. solani*. Representative fluorescence fields were chosen from at least three independent plants. **(I,J)** Fluorescence microscopy of inoculated SA-primed leaves showing scant hyphal growth at the same time point (arrow shows right angle branching with septa typical for *R. solani*).

In the previous experiment, since the *R. solani* sclerotia showed least growth on SA pre-treated leaves, we wanted to observe in greater detail the behavior of sclerotia and the emerging hyphae on SA-primed leaves using SEM and confocal microscopy (Figure 3). At 24 hpi on the control sets, numerous infection hyphae had emerged from the sclerotia (Figure 3A). The lateral branches stayed in intimate contact with the host leaf surface and formed hyphal aggregation at places shown by arrows in Figure 3A, giving rise to infection cushions. In contrast, the sclerotia on SA pre-treated leaves showed only a few emerging hyphal strands with complete absence of any aggregation (Figure 3B). At 48 hpi, the infection hyphae already formed infection cushions on the control leaves (Figures 3C,E), while on SA-primed leaves, the hyphae grew in straight lines avoiding intimate contact with the leaf surface (Figure 3D) with rare hyphal aggregates (Figure 3F). Fluorescence microscopy of control and SA-primed leaves infected with *gfp* transformed *R. solani* at 24 hpi showed typical right angles branching of hyphae with septa near the branch points (Figures 3G–J). The hyphae formed dense mats with profuse branching on the control leaves while showing scant growth on the SA-primed leaves (Figures 3G–J).

### Hyphae of *R. solani* Avoided Close Interaction With the Leaf, Stem, and Root Surface and Trichomes of Primed Plants, Showing Less Invasion of Underlying Tissue, Especially in Salicylic Acid-Primed Plants

**FIGURE 4:**
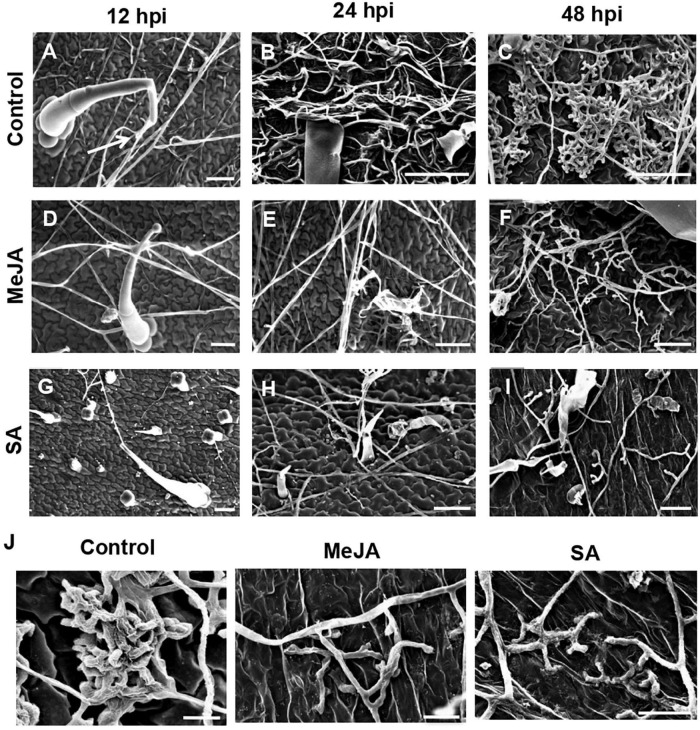
Scanning Electron Microscopy to observe differential behavior of *R. solani* hyphae on the surface of primed and control leaves, showing interaction with trichomes and formation of infection cushions during early stages of infection. **(A)** Control leaf showing close association of hyphae with leaf trichome at 12 hpi (Arrow) **(B)** Hyphae making intimate contact with the control leaf surface forming network of hyphae at 24 hpi. **(C)** By 48 hpi, coalesced infection cushions cover most of the leaf surface in control leaves. **(D)** In the MeJA-primed leaves, the hyphae avoid contact with leaf surface, interacting only with the trichomes at 24 dpi. **(E)** On MeJA-primed leaves, the hyphae show sparse growth with less hyphal branching and **(F)** initiation of infection cushions with clubbed bifurcating hyphae, is delayed to 48 hpi. **(G–I)** The SA-primed leaf surface showing least hyphal growth with superficial hyphal contact with leaf surface and no infection cushion even at 48 hpi. Bar = 50 μm. **(J)** Close-up views of infection cushions or hyphal aggregations in respective experimental sets at 48 hpi. Bar = 20 μm.

During the establishment phase of the fungus on the control leaf surface, the hyphae interacted with the trichomes of the control leaves from 12 hpi (Figure 4A) and formed a hyphal network closely pressed to the epidermal terrain by 24 hpi (Figure 4B). By 48 hpi, the pathogen formed infection cushions that coalesced to form larger masses on the control leaves (Figures 4C,J). On the MeJA-primed leaves, the hyphae showed superficial interaction with the trichomes and avoided close contact with the leaf surface, only forming hyphal aggregated at places at 48 hpi (Figures 4D–F,J). The hyphae on the SA-primed leaves showed no interaction with trichomes at 24 hpi and showed least hyphal growth even at 48hpi (Figures 4G–J).

**FIGURE 5:**
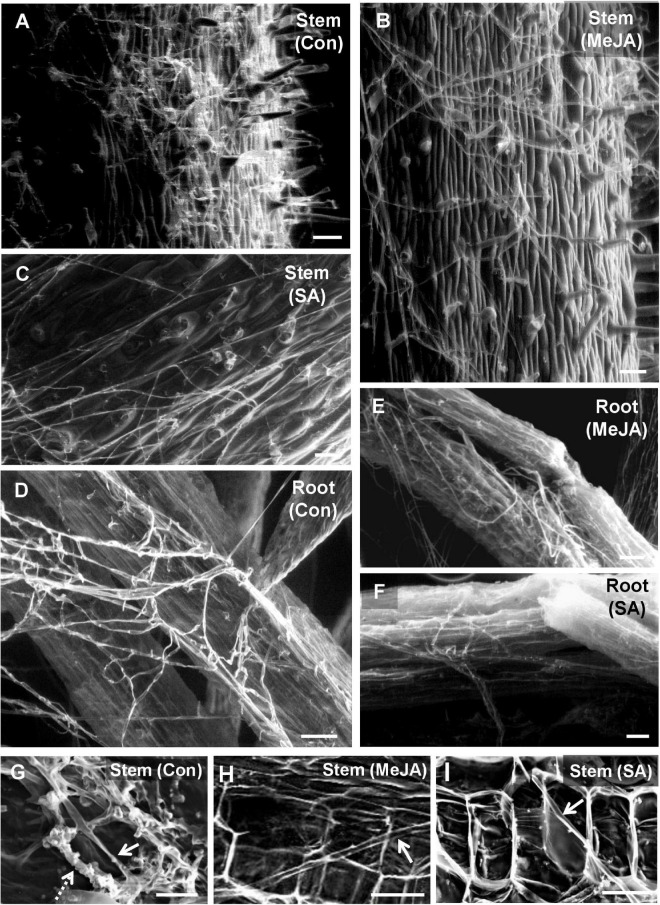
Scanning electron microscopy of tomato stem and root surfaces showing differential behavior of *R. solani* hyphae at 2 days post-inoculation. **(A)** Profuse growth of hyphae covering the stem surface of control plants at 48 hpi forming tangled mats at places. **(B)** Significantly less growth of hyphae on the stem surface of MeJA pre-treated plants showing loose hyphal network. **(C)** SA pre-treated stem surface showing least hyphal load with the hyphae running straight without forming hyphal network **(D)** Roots of control plant showing a significant amount of thick hyphal growth on the surface, whereas a minimal growth of hyphae is found on MeJA-primed roots **(E)**, and even less on SA-primed roots **(F)**. Bar = 100 μm **(G)** Regular occurrence of intracellular (solid arrow) and thick bunches of intercellular hyphae outlining the individual cells (dashed arrow) within the stem cortical tissue as seen in the transverse section through the stem at 48 hpi. **(H,I)** Rare presence of thin intracellular hyphae (arrow) in certain regions of stem cortical tissue in MeJA **(H)** and SA **(I)**-primed pants, though at most regions, in the cortical tissues there was absence of any hyphae at 48 hpi. Bars = 50 μm.

The above behavior of the pathogen on the leaf surface was also reflected on the stem and root surfaces. The dense, profuse fungal growth on the surface of the stem in the control plants showed close interaction with the stem trichomes (Figure 5A). On MeJA pre-treated leaves, the hyphal growth was reduced to a loose dispersed network with superficial interaction with stem trichomes (Figure 5B). In the case of SA pre-treated plants, an interesting behavior was observed. Here, the mycelium strands grew parallel to each other without producing many lateral branches (Figure 5C). It appeared like the pathogen in this particular phytohormone treatment and tried to grow forward without branching. Similar behavior was also noticed in the case of roots (Figures 5D–F). Cross-sections through stems of control plants showed intracellular growth of hyphae (Figure 5G, solid arrow) and thick bunches of hyphae growing intercellularly outlining the cortical cells (dashed arrow). The primed stems showed thin hyphae with intracellular growth at places in the cortical tissues (Figures 5H,I).

### Confocal Laser Scanning Microscopy of Primed and Control Tomato Leaves and Stems Infected With *R. solani* Transformed With Green Fluorescence Protein Gene

**FIGURE 6:**
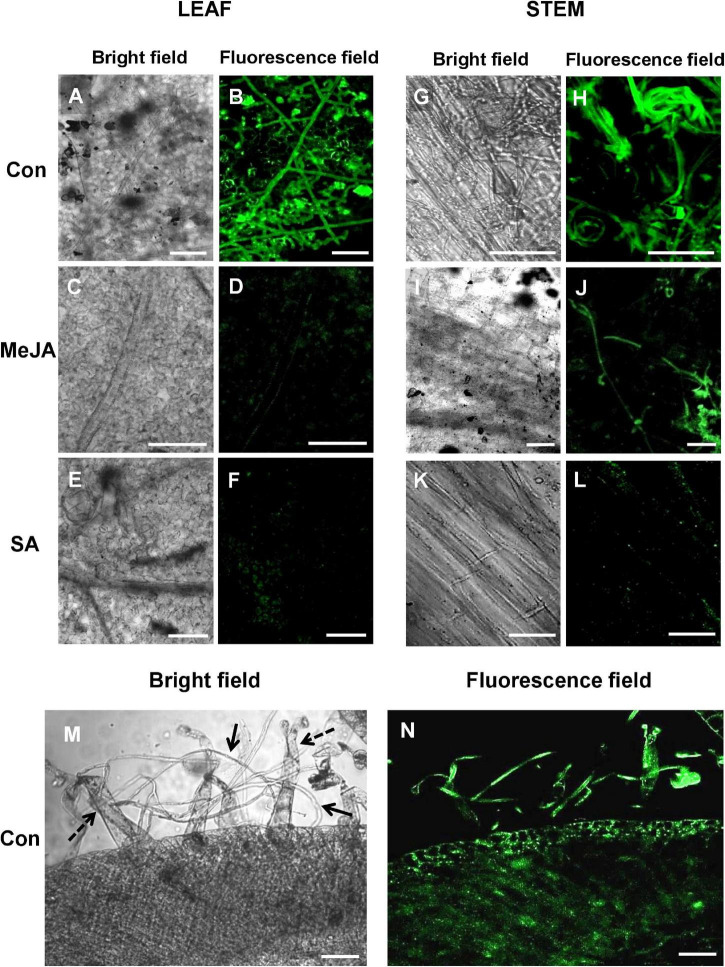
Confocal Laser Scanning Microscopy (CLSM) of *R. solani* transformed with green fluorescence protein gene (*gfp*) on infected tomato leaves and stems showing avoidance of primed plants, especially SA-primed plants at 24 hpi. **(A,B)** CLSM of control tomato leaves showing profuse hyphal growth with infection cushions in close contact with the leaf surface. **(C–F)** JA and SA-primed leaves show significantly less growth of hyphae at 24 hpi. **(G–L)** Confocal microscopy of stems of primed and control plants infected with transformed *R. solani* showing least growth on SA-primed stem. **(M,N)** Transverse section of infected control stem showing significant interaction of hyphae (solid arrows) with stem-surface trichomes (dashed arrows) and infection within tissue at 24 hpi. Representative fluorescence fields were chosen from at least three independent plants. Bar = 50 μm.

Confocal laser scanning microscopy (CLSM) of leaves and stems of plants inoculated with transformed *R. solani* showed significant establishment of the fungus on the control leaves and stems, less infection in the MeJA-primed plants, and least in the case of SA-primed plants (Figures 6A–L). As seen with SEM, confocal microscopy of cross-section of the control stem revealed significant interaction of fungal hyphae with the trichomes and invasion of underlying tissues (Figures 6M,N).

### Salicylic Acid Pretreatment Contributed to Overall Reduced Disease Severity and the Least Disease Index Compared to Methyl Jasmonate-Primed and Control Plants

**FIGURE 7:**
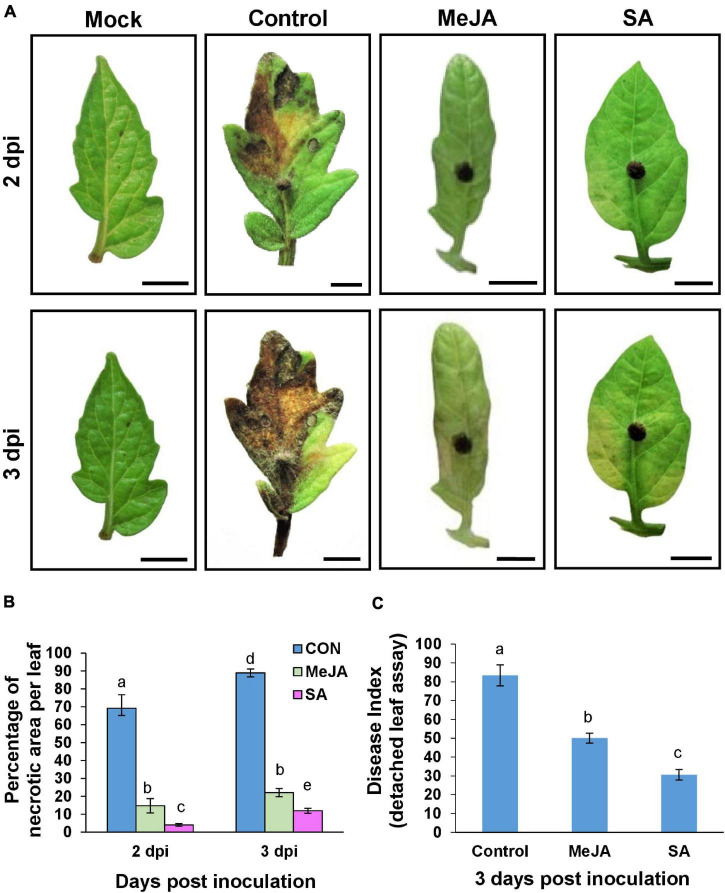
Detached leaf assay showing priming with phytohormones contributed to reduced disease severity and less disease index with SA pre-treatment being more effective than MeJA. **(A)** Comparison of development of necrotic lesions at 2 and 3 dpi on control and phytohormone treated tomato leaves after inoculation with *R. solani*. At 2 dpi the control set leaves develop significant necrotic lesions whereas MeJA and SA-primed leaves remained almost as fresh as the unprimed, uninoculated (mock) leaves. Control leaves get almost entirely necrosed by 3 dpi while MeJA and SA-treated leaves show significantly less necrosis. **(B)** Graph showing comparison of the percentage of necrotic lesion on leaves from the above experiment. **(C)** Disease index at day 3 showing that SA pretreatment results in least disease index. Bars represent mean ± S.E.M of three independent experiments with three replicates. Different letters indicate significant differences among treatments at *p* < 0.05, according to Duncan’s multiple range test.

**FIGURE 8:**
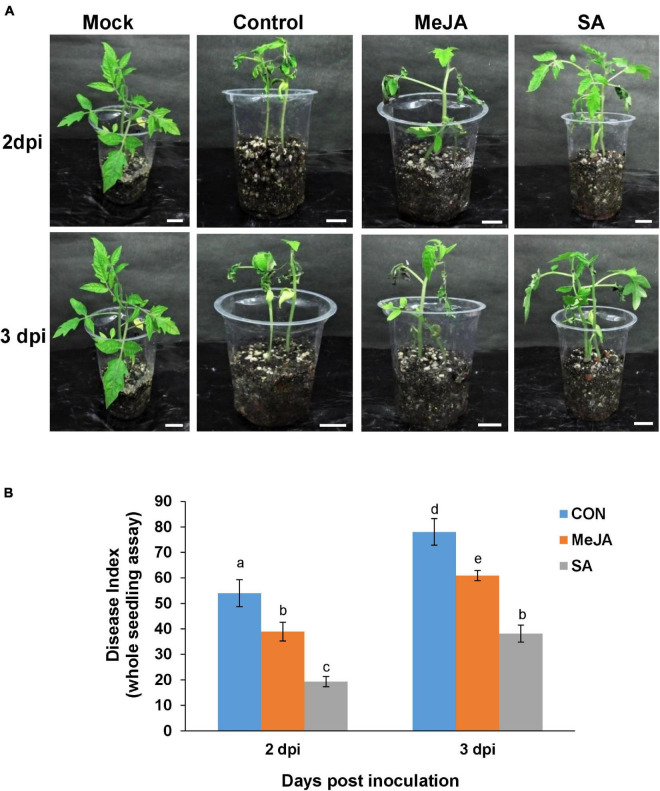
Whole seedling assay showing disease incidence by *R. solani* on phytohormone primed plants compared with control. **(A)** MeJA pre-treated plants showed less severe symptoms than the control and but more symptoms than SA-treated ones at 2 and 3 days post-inoculation, in the order Control > MeJA > SA. **(B)** Comparative graphical representation of disease index in the different experimental sets at 2 and 3 dpi, calculated according to the published method. Bars represent mean ± S.E.M of four independent experiments. Different letters indicate significant differences among treatments at *p* < 0.05, according to Duncan’s multiple range test. Bars = 2 cm.

The avoidant behavior of the pathogen on the primed host surface was reflected in the reduction of overall disease severity, especially in the SA-primed plants. Disease Index was measured by detached leaf assay (Figure 7) and by whole seedling assay (Figure 8). Phytohormone treatment significantly delayed the onset of symptoms which was evident even from the first-day post-inoculation. Necrotic symptoms started to be visible at 2 dpi in the case of control plants (Figure 7A), but these lesions quickly increased in size so that by 3 dpi, the control leaves were almost entirely necrosed (Figure 7A). In comparison with the control, the MeJA pretreated leaves showed almost no necrotic symptoms up to 2 dpi and only on the 3rd day after inoculation, did the MeJA pretreated leaves show small necrotic spots. The SA pre-treated leaves showed yellowing at 3 dpi with very few necrotic lesions (Figure 7B). The SA-primed plants also had the least disease index (Figure 7C).

The whole seedling assay under these experimental conditions showed similar results. The MeJA pre-treated plants showed less severe symptoms than the control and more severe than SA-treated ones at the corresponding time points (Figure 8A). The severity of the disease was in the order of Control > MeJA > SA from maximum to minimum (Figures 8A,B). Overall, at 2 dpi, there was about 50% less disease index in the SA-primed leaves compared to MeJA-primed leaves and almost 40% less at 3 dpi (Figure 8B).

### The Development of Infection Cushions, Appressoria, and the Expression of the Appressorial Penetration-Associated Gene Is Influenced by the Priming Status of the Host With Salicylic Acid Priming

**FIGURE 9:**
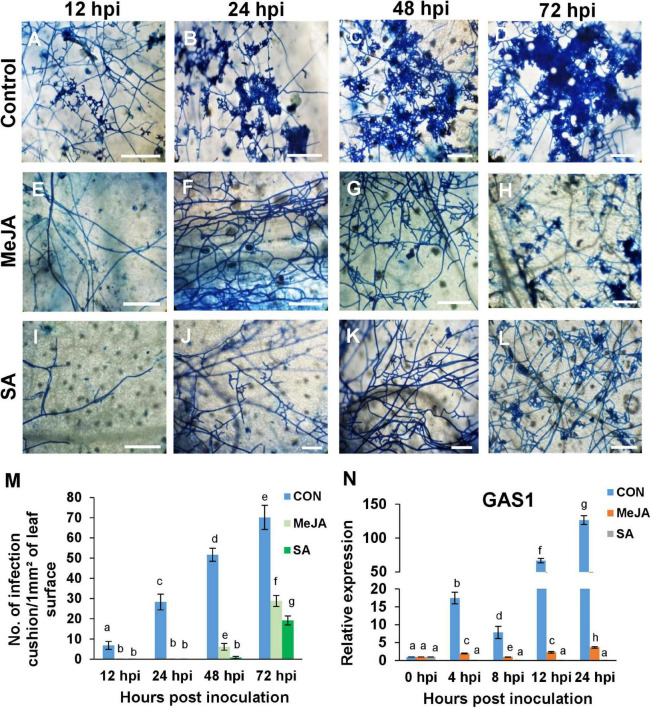
The priming status of the host has direct effect on the frequency of infection cushions, and the expression of the appressorial penetration-associated gene (GAS1) of the pathogen. **(A–D)** Development of infection cushions (seen as deep blue patches) was initiated as early as 12 h after inoculation in the control plants and progressed rapidly over time, until at 72 hpi, there was coalescence of the infection cushions. **(E–H)** MeJA priming significantly delayed this process and initiation started as late as 48 hpi. **(I–L)** Pre-treatment with SA reduced the formation of infection cushions further. At 72 hpi, SA sets showed smaller, more dispersed infection cushions than the MeJA sets. **(M)** Comparison of the density of infection cushions between the different sets. **(N)** Relative expression of appressorium-associated protein gene GAS1, showing significantly high transcript level in the control compared to the phytohormone treated sets, over a time course, with the SA sets showing least expression. Bars represent mean ± S.E.M of three independent experiments with three replicates. Different letters indicate significant differences among treatments at *p* < 0.05, according to Duncan’s multiple range test. Bar = 100 μm.

To see if the initiation of the infection cushions and appressoria was altered by the priming status of the host, the initial stages of establishment of the pathogen were observed over three days. Development of lobate appressoria started as early as 12 hpi in the case of control leaves (Figure 9A). It then advanced rapidly over time and ended up in the formation of dense coalesced masses of hyphae by 72 hpi (Figure 9D). On the surface of MeJA and SA pretreated leaves, however, the pathogen could not initiate the formation of infection structures up to 48 hpi, and infection cushions started to develop as late as 72 hpi (Figures 9E–L). The frequency of infection cushion was quantified as the number of infection cushions developed per unit area on the leaf surfaces. The development of infection cushion on the control was three times greater than that of the two phytohormone pretreated leaves at 72 hpi (Figure 9M). When compared between the two primed leaves, SA pretreatment supported approximately 25% less infection cushions compared to the MeJA pretreated leaves.

To confirm the effect of pre-treatment on the formation of infection cushion, we observed the expression of the Appressorial Penetration-associated gene (*GAS1*) in the pathogen. This gene was expressed significantly in the control plants from 4 hpi onward, whereas it was significantly less in MeJA and least in the SA-primed background (Figure 9N). This low expression of the *GAS1* gene directly correlated with a much lower number of infection cushions in MeJA and SA-primed plants than control at all time points (Figure 9M).

### Salicylic Acid Priming Enhanced Callose Deposition in Response to Infection in Early Phases While Methyl Jasmonate Enhanced It in the Later Phases of Infection

**FIGURE 10:**
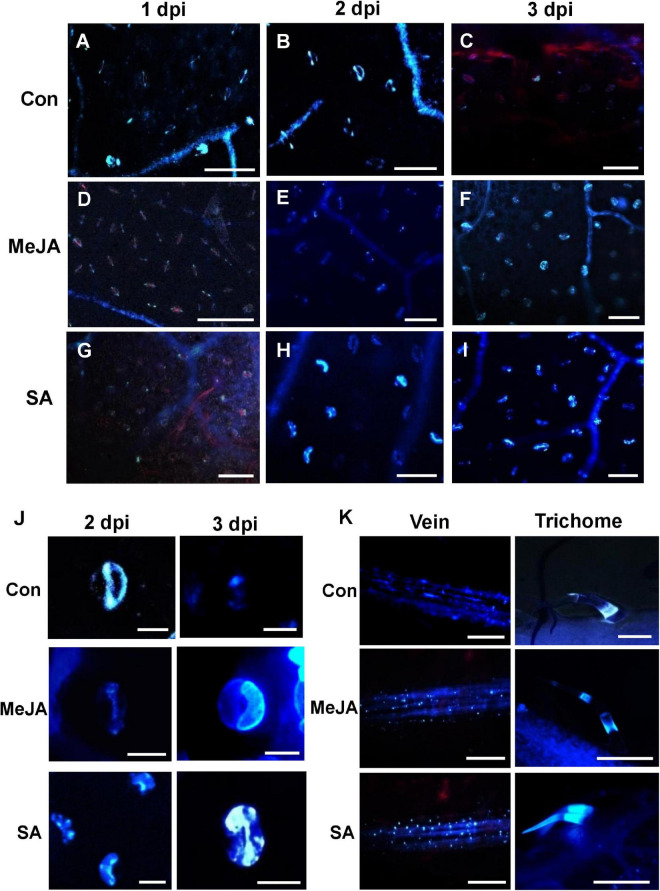
Comparison of callose deposition on the leaf surface, veins, stomata, and trichomes show that SA priming enhanced callose deposition in response to infection in the early phases while MeJA enhanced in the later phases. **(A–C)** The control plants show early callose deposition on leaf surface, veins, and stomata over the first two days of infection, while on the third day callose is degraded due to necrosis. **(D–F)** Deposition of callose in the veins, stomatal guard cells in MeJA-primed leaves with more prominent deposition starting on the 3 dpi **(G–I)** Callose deposition in the SA-primed leaves started on the second day and was significantly more than that of MeJA-primed plants. Three images were analyzed from four individual replicates. Bar = 50 μm. **(J)** Enlarged view of the stomata at 2 and 3 dpi. In the control leaves, only one of the guard cells of each stoma fluoresced on 2 dpi and then by third day it diminishes. In MeJA, no fluorescence on 1–2 dpi but both guard cells fluorescence on 3 dpi. In SA-primed leaves, at 1 dpi there is no fluorescence, at 2 dpi only one guard cell fluorescence and in the following day both of the guard cells fluoresce. **(K)** Anniline blue fluorescence of the veins and trichomes at 2 dpi shows less callose deposition in the control compared to the phytohormone primes leaves. Bar = 300 μm.

Callose was found to be deposited in several parts of primed tomato leaves after *R. solani* infection. Stomata, veins, and trichomes were most frequently found to be the sites of greater deposition (Figure 10). We observed interesting differences in callose deposition in response to pathogen under different priming status of the host. The stomatal fluorescence was more intense in the case of SA pretreated leaves compared to control leaves and MeJA-primed leaves (Figures 10A–I). In MeJA-primed leaves, callose deposition was prominent only at 3 dpi (Figure 10F) where both of the guard cells of each stoma fluoresced (Figure 10J). At 1 dpi, in control and SA pretreated leaves, most guard cells fluoresced only at the two poles of the stoma, which increased considerably with increasing time (Figure 10J). Following this stage, only one of the pair of guard cells in each stoma fluoresced in both control and SA plants at 2 dpi. At 3 dpi, in SA-primed leaves, the callose deposition spread to the other guard cell, making a completely fluorescing stoma. In the control sets, by 3 dpi, the leaves showed necrosis and no stomatal fluorescence was detectable (Figure 10J).

More deposition of callose was observed on the veins and trichomes in the case of SA-primed leaves compared to the MeJA and Control leaves. In the MeJA pre-treated leaves, the fluorescence was intermediate (Figure 10K).

### Salicylic Acid Priming Enhanced Phenol Deposition in Earlier Phase While Methyl Jasmonate Enhanced It in the Later Phase of Infection

**FIGURE 11:**
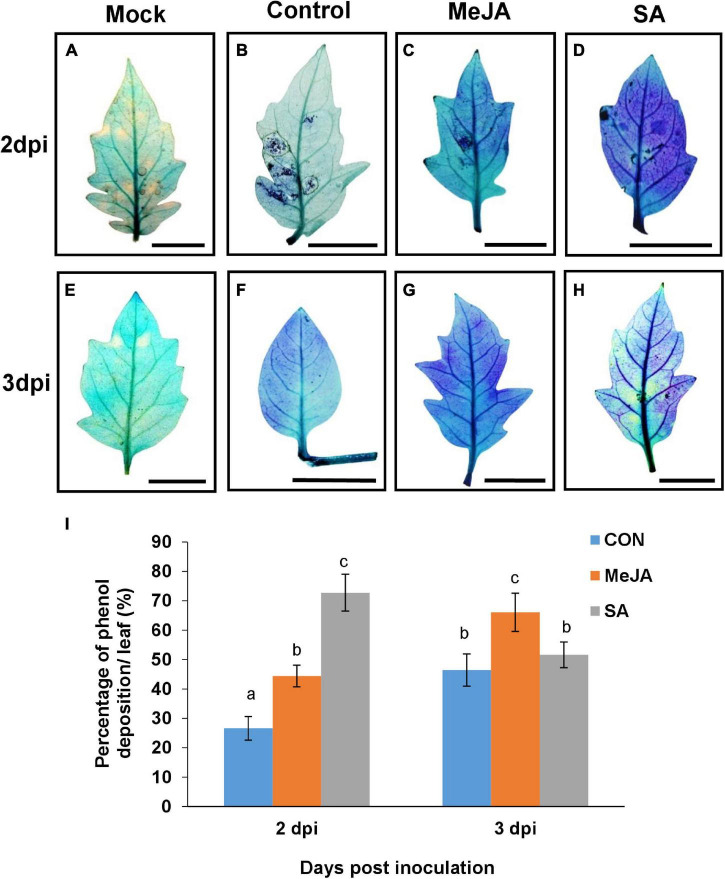
Accumulation of phenol detected by toluidine blue shows that SA priming resulted in more deposition in earlier phase of infection while MeJA priming in the later phases. **(A–H)** The development of blue color indicate the amount of phenol in leaves of different sets of control and primed leaves at 2 and 3 days post infection. **(I)** Graph representing the comparison of the above result. Bars represent mean ± S.E.M of six independent experiments. Different letters indicate significant differences among treatments at *p* < 0.05, according to Duncan’s multiple range test. Bar = 30 mm.

Secondary metabolites are an important component of inducible defense, and phytohormone priming is known to induce their production. These parameters were compared in the MeJA and SA-primed backgrounds after infection to understand why SA imparted better tolerance to the necrotroph, contrary to expectation. Differential accumulation of phenol was recorded in response to *R. solani* infection on the leaves of control and phytohormone treated leaves *via* toluidine blue assay. SA pretreatment was found to be highly inducive to the increase of the total phenol content in leaves in the early phase of infection at 2 dpi, showing significantly more phenol accumulation than the control and MeJA pre-treated leaves (Figure 11). Interestingly, in the case of MeJA pretreatment, the accumulation was less than that of SA at 2 dpi but increased in the later stages of infection at 3dpi (Figure 11).

### Salicylic Acid Priming Induced Polyphenolics and Their Genes in the Earlier Phase of Infection While Methyl Jasmonate Induced These in the Later Phase

**FIGURE 12:**
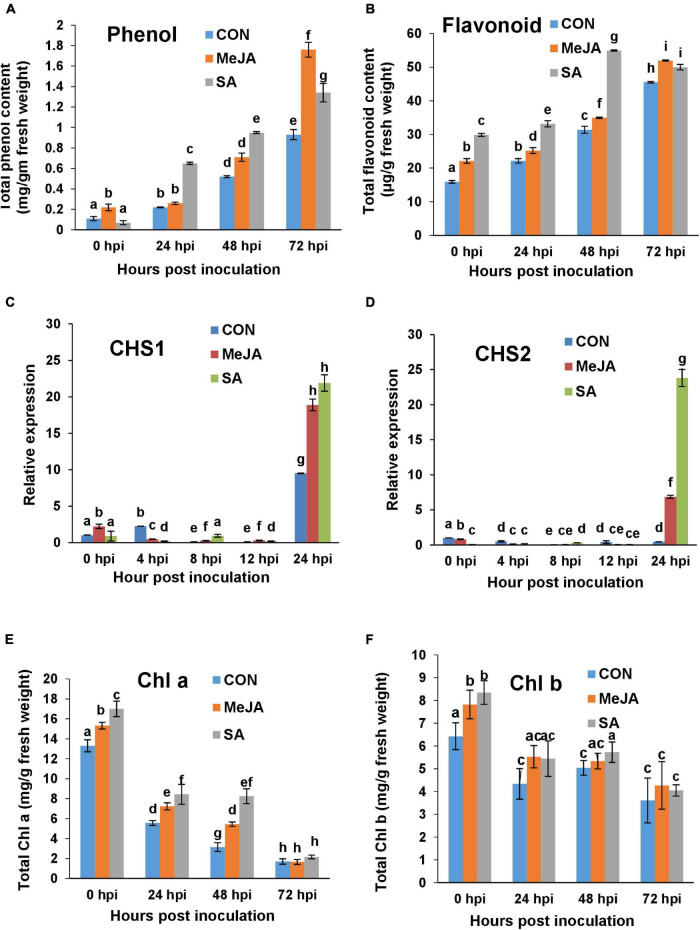
Comparison of accumulation of polyphenolics, induction of their genes, and chlorophyll content in the control and phytohormone primed plants during *R. solani* pathogenesis. **(A)** Total phenol content in different sets over a time course. **(B)** Total flavonoid content. **(C,D)** Quantitative real-time PCR analysis of the expression of Chalcone synthase genes *CHS1* and *CHS2* respectively over a time course. **(E,F)** Chlorophyll a (Chl a) and Chlorophyll b (Chl b) content respectively showing least degradation after infection in SA-primed leaves compared to Control and JA-primed leaves. Bars represent mean ± SEM of three independent experiments with at least three individual plants. Different letters indicate significant differences among treatments at *p* < 0.05, according to Duncan’s multiple range test.

Secondary metabolites, especially polyphenolic compounds, are an integral part of the plant basal defense. The total phenol content in tomato plants during *R. solani* pathogenesis assayed through Folin Ciocalteau method remained almost unaltered up to 24 hpi in control and MeJA-primed plants but was elevated in SA-primed plants (Figure 12A). As with toluidine blue assays, the order of phenol deposition was SA > MeJA > control at 24 and 48 hpi. However at 72 hpi, MeJA pretreatment was found to be highly inducive to total phenol content, and the pattern is altered to MeJA > SA > Control. In the case of flavonoid content, the difference was in the order SA > MeJA > Con for almost all time points, although the difference was more pronounced in 48 hpi (Figure 12B).

The above result of enhanced polyphenol content was corroborated by altered regulation of two key flavonoid synthesizing gene, namely, Chalcone biosynthetic genes *CHS1* and *CHS2. CHS1* and *CHS2* did not significantly change at early time points of pathogenesis and were only upregulated at 24 hpi. At 24 hpi, the expression of these genes was significantly more in SA pre-treated plants than MeJA (Figures 12C,D). The differences were nearly two-fold between SA and MeJA treatments in the case of *CHS2* (Figure 12D).

### Both Methyl Jasmonate and Salicylic Acid Priming Reduced Chlorophyll Degradation During Pathogenesis, With Salicylic Acid Offering Better Protection

Chlorophyll content was found to be significantly affected after infection. Chlorophyll was degraded vigorously with the progresses of pathogenesis in control plants. However, both MeJA and SA priming successfully limited the extent of degradation of Chl a throughout the course of pathogenesis (Figure 12E). Interestingly Chl a was least degraded in SA-treated plants in the early time points of infection at 24 and 48 hpi, which evened out at 72 hpi as infection set in for all three sets. Chl b was also degraded gradually after infection. However, it did not differ significantly between sets (Figure 12F).

### Salicylic Acid Priming Offered Better Protection Against Reactive Oxygen Species During Infection Than Methyl Jasmonate Priming

**FIGURE 13:**
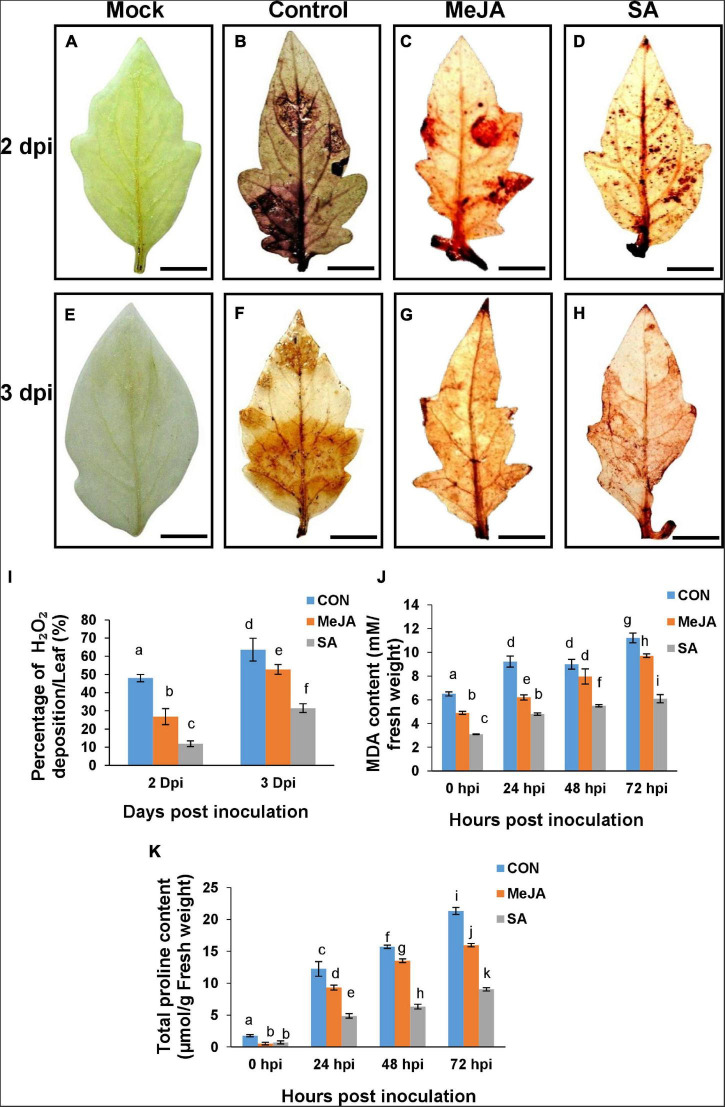
Detection of reactive oxygen species (ROS) through Diaminobenzinidine (DAB) staining and accumulation of proline, show SA priming offered better protection against ROS during infection than MeJA priming. **(A,E)** Mock unprimed and uninoculated leaves showing the background color developed by the stain. **(B,F)** Control leaves showing deposited H_2_O_2_ generated after pathogen infection at 2 and 3 dpi. **(C,D,G,H)** presence of ROS on the MeJA and SA pre-treated leaves, respectively. **(I)** Quantitative bar graph of the above result. **(J)** MDA content and **(K)** total proline content through the experimental time course. Bars represent mean ± S.E.M of six independent experiments. Different letters indicate significant differences among treatments at *p* < 0.05, according to Duncan’s multiple range test. Bar = 10 mm.

Reactive oxygen species (ROS) is one of the key contributing factors in determining the successful establishment of the pathogen. The amount of ROS was determined by DAB staining, which was detected as brown patches on the leaves. Control leaves had significantly higher amount of H_2_O_2_ at all time points compared to primed leaves as expected (Figure 13). Surprisingly, MeJA pre-treated leaves showed almost double the amount of H_2_O_2_ than that of the SA pre-treated leaves at different time points (Figure 13I), indicating that SA pretreatment created less stress and offered better protection against the pathogen than MeJA. The correlation of H_2_O_2_ content with degradation of chlorophyll and the pathogen as the source of the excess H_2_O_2_ have been discussed in later sections.

Significant reduction of oxidative burst by phytohormone priming was also validated by suppression of lipid peroxidation (MDA) (Figure 13J) and proline content in the primed sets (Figure 13K). The results were similar to H_2_O_2_ accumulation. Evidently, salicylate pretreatment can better limit the ROS production stimulated by *R. solani* infection than Jasmonate pre-treatment which also explains the overall least disease index in SA-primed plants.

### Altered Defense Gene Regulation, in Response to Pathogen After Phytohormone Priming, Indicate Salicylic Acid-Mediated Signaling in the Early Phases, and Methyl Jasmonate-Mediated Signaling in the Later Phases of Infection

To date, there is no in-depth study on the MeJA and SA-mediated defense gene regulation during post-necrotrophic infection in plants primed with SA compared with MeJA priming. To investigate the basis of differential defense responses to *R. solani* when primed with SA and MeJA, we selected a total of 10 marker genes from salicylate and jasmonate signaling cascades. Their expression profiles were monitored over a time course during pathogenesis in these primed backgrounds.

### Regulation of Jasmonate Signaling Pathway

We included the F-box protein *CORONATINE INSENSITIVE 1* gene (*COI1*), the repressor protein *JASMONATE ZIM DOMAIN* gene (*JAZ*), along with Allene Oxide synthase gene (*AOS*), a leucine-rich repeat systemin receptor kinase (LRR-RK) gene (*SR 160*), and a protease inhibitor gene (*PIN II*) as important components of MeJA signal transduction pathway.

**FIGURE 14:**
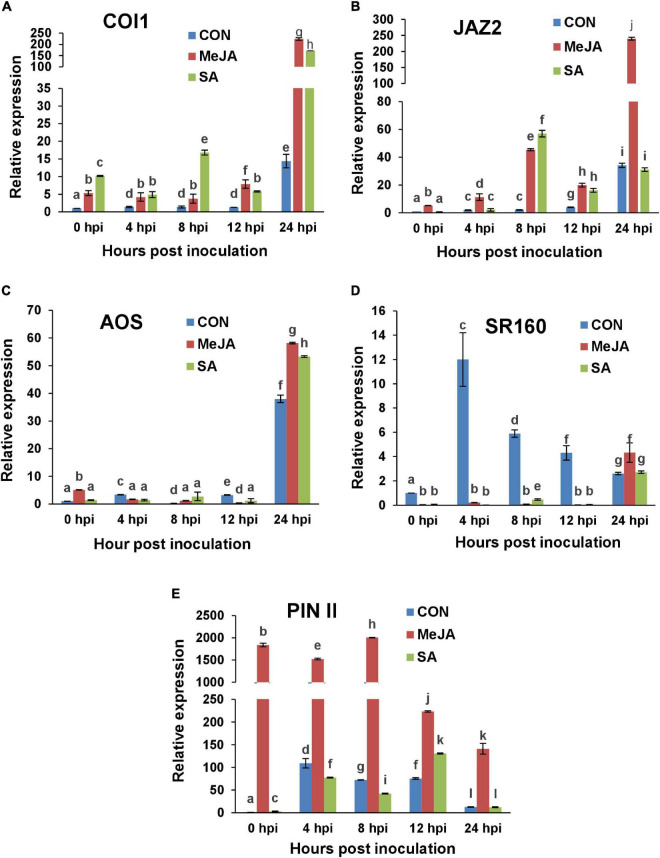
RT-qPCR analysis of the expression of Jasmonate signaling marker genes in primed and control plants post-infection with *R. solani*. Graphs of Quantitative real-time RT-PCR analysis of genes over a time course in the first 24 h after infection: **(A)** *CORONATINE INSENSITIVE 1* (*COI1*), **(B)** Jasmonate zinc-finger inflorescence meristem (ZIM) domain (*JAZ2*), **(C)** Allene oxide synthase (*AOS*), **(D)** Systemin receptor SR160 (*SR160*), **(E)** Proteinase inhibitor II (*PIN II*). Bars represent standard error (SE) of the mean (*n* = 3). Different letters indicate significant differences among treatments at *p* < 0.05, according to Duncan’s multiple range test.

There was considerable upregulation of the *COI1* gene within 24 h of spraying of phytohormones, i.e., at 0 hpi, with SA priming resulting in nearly double *COI1* transcript levels compared to MeJA priming (Figure 14A). After SA pre-treatment, the infection resulted in two peaks of *COI1* transcripts at 8 and 24 hpi while in MeJA-primed plants, *COI1* showed a steady rise after an initial dip at 4 hpi, which is 28 h post priming. The *COI1* transcripts remained high at 48hpi, then went down at 72 hpi near to the control level (Supplementary Figure 1A).

Similar biphasic upregulation was observed in the case of *JAZ* in SA-primed plants (Figure 14B). *JAZ* was significantly upregulated at 24 hpi in the MeJA-primed plants coinciding with *COI1*, likely as a means to regulate JA signaling at this time. Interestingly, in SA-primed plants, although *COI1* was significantly upregulated at 24 hpi, the repressor, *JAZ*, was not (Figures 14A,B). Moreover, this low level of expression of JAZ was maintained in the next two time points of 48 and 72 hpi in the SA-primed plants (Supplementary Figure 1B). Hence, contrary to expectation, this shows that *R. solani* infection after priming with exogenous SA induced the JA marker genes even more than priming with MeJA itself in the early time points post-infection, i.e., in the first eight hpi. MeJA priming, on the other hand, induced the JA marker genes more than SA priming, but only at 12 hpi onward. This is contrary to expectation because *R. solani* is considered as a necrotroph and not a hemibiotroph. This will be discussed in greater detail in the next section. Overall, these results indicate that along with the conventional *COI1* dependent activation of jasmonate signaling against the invading pathogen, SA priming played an even greater role in the early time points post-infection than MeJA priming. This explains why the disease index was least for SA-treated plants compared to the control and MeJA pre-treatment (Figures 7, 8).

Jasmonic acid (JA) biosynthesis gene *AOS* was upregulated at 24 hpi in all experimental sets. However, *AOS* transcript levels were highest in the case of jasmonate priming at that time point, in the order MeJA > SA > Con sets (Figure 14C). At 48 hpi, *AOS* transcript level was doubled in MeJA-primed plants which declined sharply in the next time point (72 hpi). Contrastingly *AOS* induction decreased in both control and SA-primed plants in the next two time points (Supplementary Figure 1C).

One interesting fact was observed in the case of expression of the early gene Systemin Receptor 160 (*SR160*) (Figure 14D). In the control plants, *SR160* was upregulated within 4 hpi and then, the transcript level gradually fell. Contrastingly, exogenous application of MeJA and SA was able to delay the upregulation of the Systemin receptor gene till 24 hpi. At the next time point of 48 hpi, *SR160* transcript level peaked in MeJA-primed background (Supplementary Figure 1D). One explanation would be that the phytohormone pre-treatment delayed the disease progression and hence the release of systemin from infected tissues was automatically delayed. Most importantly, SA pre-treatment showed significantly less SR160 transcript levels even at 24 hpi compared to MeJA-primed plants, corroborating the fact that SA priming contributed to greater tolerance to the pathogen than MeJA priming.

A protease inhibitor gene (*PIN II*) was significantly upregulated in the MeJA-primed plants from 0 hpi, i.e., from 24 h after priming (Figure 14E). The transcript level remained significantly elevated up until 8 hpi and then was reduced from 12 hpi onward. In the control and SA-primed plants, *PIN II* was upregulated during 4-12 hpi although not nearly as high as the MeJA-primed plants. In the later time points, the expression of this gene remained at levels nearer to that of the control in all sets (Supplementary Figure 1E).

### Regulation of Salicylate Signaling Pathway

In general SA-mediated defense signaling is thought to be directed against hemibiotrophic and biotrophic pathogens. To compare defense response against *R. solani* after priming with SA and MeJA, we included five SA pathway marker genes, including two major SA biosynthetic genes, *viz.*, Isochorismate synthase (*ICS*), Phenylalanine ammonia lyase 5 (*PAL5*), along with important components of SA signal transduction pathway such as BA/SA carboxyl methyltransferase 1 gene (*BSMT1*), Pathogenesis-related protein-1a gene (*PR1a*), and the Phytoalexin-deficient 4 gene (*PAD4*).

**FIGURE 15:**
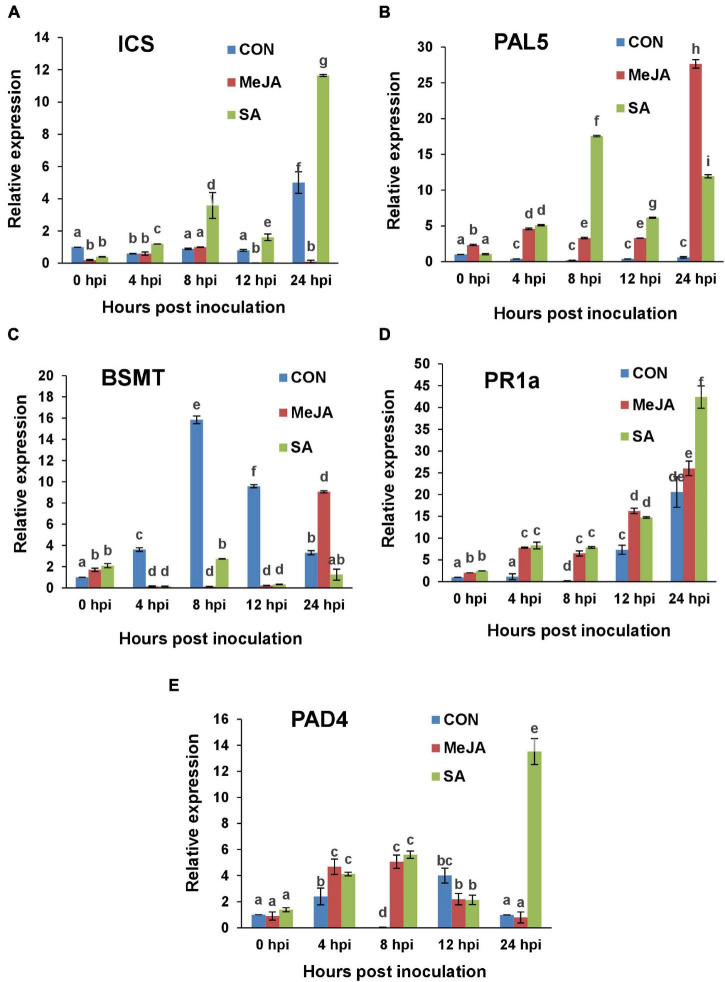
RT-qPCR analysis of the expression of Salicylate signaling marker genes in primed and control plants post infection with *R. solani*. Graphs of Quantitative real-time RT-PCR analysis of genes over a time course in the first 24 h after infection: **(A)** Isochorismate synthase (*ICS*), **(B)** Phenylalanine ammonia lyase 5 (*PAL5*), **(C)** Salicylate/benzoate carboxyl methyltransferase (*BSMT*), **(D)** Pathogenesis-related protein-1a (*PR1a*), **(E)** Phytoalexin-deficient 4 *(PAD4*). Bars represent standard error (SE) of the mean (*n* = 3). Different letters indicate significant differences among treatments at *p* < 0.05, according to Duncan’s multiple range test.

It was observed in the case of SA pre-treated plants that *ICS* (Figure 15A) and *PAL5* (Figure 15B) were both activated during the early time points of pathogenesis, especially at 8 hpi. Later on, at 24 hpi, the SA production was shifted mainly through the isochorismate pathway in the SA-primed plants showing a high *ICS* transcript level, which gradually went down in the 48 and 72 hpi (Supplementary Figure 2A). Interestingly, in MeJA pre-treated background, the plants relied solely on the *PAL5* pathway for SA biosynthesis as evidenced by its induction throughout, especially high induction at 24 hpi (Figure 15B), while avoiding the *ICS* pathway as there is little or no expression of this gene in MeJA-primed plants (Figure 15A). *PAL5* subsequently went down in MeJA-primed plants. In the SA-primed plants, *PAL5* rose till 48 hpi before going down at 72 hpi (Supplementary Figure 2B).

The expression of the SA regulatory gene *BSMT* (required to mobilize SA out of the site) in the control plants showed a peak at 8 hpi after that it declined gradually till 24 hpi (Figure 15C). For SA-primed plants, *BSMT* remained low in its expression until at 72hpi in which it reached control levels. In MeJA-primed plants BSMT showed a sharp rise at 24 hpi likely to mobilize SA out of the site as JA defense sets in at this time point. In the next two days BSMT expression declined gradually in the MeJA-primed plants (Supplementary Figure 2C).

One of the key marker genes for SAR is pathogenesis-related proteins (PR) which is not only diverse in their genetic constituents and functions, but also in its response to different components as well as different pathogens. In this study, the acidic PR protein gene, *PR1a* has been found to be induced gradually in the primed plants while there was less expression in the control plants. The *PR1a* expression showed a steady gradual increase in response to infection in SA-primed plants and became significantly higher than MeJA-primed plants at 24 hpi (Figure 15D) as expected. Over the next two days, *PR1a* expression level gradually went down in the SA-primed plants, whereas it was significantly low for control and MeJA-primed plants (Supplementary Figure 2D). In the case of *PAD 4*, there was many-fold increase in the SA-primed plants at 24 hpi compared to both MeJA and control (Figure 15E). This elevated *PAD4* level was maintained at 48 hpi also and then started declining in the SA-primed plants (Supplementary Figure 2E).

### Quantification of Salicylic Acid and Jasmonate Showed More Salicylic Acid in the Early Phases and More Jasmonate in the Later Phases Under Both Types of Priming

Time course quantification of phytohormones in plants after *R. solani* infection showed a spike in SA content to about 15,000ng/gm fresh weight of leaves at 24 hpi in the SA-primed plants (Supplementary Figure 3A). In the next phases of up to 72 hpi, a decline in the SA content was seen in these plants. The JA content gradually increased and reached a peak amount of about 5,200ng/gm at 48 hpi in these SA-primed plants (Supplementary Figure 3A). On the other hand, although a similar trend was seen in the case of the JA-primed plants, the peak of SA reached only 5,300 ng/gm at 24 hpi which is only 1/3 of the amount seen in the SA-primed plants (Supplementary Figure 3B). There was considerable increase in JA content right from 0 hpi (i.e., 24 h after priming), reaching a peak at 48 hpi with a JA content of 8,500ng/gm in the JA-primed plants. So, in both SA and JA-primed plants, there was a peak in SA in early phase of infection (24 hpi) and a later peak in JA at 48 hpi. However, the SA peak in the SA-primed background was three times more than that of JA-primed plants (Supplementary Figures 3A,B). This indicates the importance of increased amounts of SA in the early phases of *R. solani* infection, even more so than the amount of JA in the later phases, which was likely a decisive factor in the outcome of the disease. In the infected control plants, the amounts of both SA and JA were significantly less in all-time points, although the profile was similar. The mock plants (no priming, no infection) did not exhibit significant differences throughout the time-course in terms of SA and JA content, which was less than even the control infected plants.

Quantification of SA and JA was also done on plants that were primed but not infected to see the effect of priming alone (Supplementary Figure 4). Priming with SA (without infection) contributed to elevated level of SA content in leaves and it reached approximately 5,000 ng/gm of fresh weight 24 hours post-priming. Subsequently, the SA content gradually went down (Supplementary Figure 4A). Priming with JA (without infection) also contributed to increase in SA level, although it was significantly less than that of SA priming. In the case of priming with JA, an initial sharp rise in JA was observed amounting to approximately 6,800 ng/gm, but at later stages, there was rapid decline (Supplementary Figure 4B). The SA priming also resulted in the increase in the JA content, although it was not nearly as high as JA priming did.

### The Phytohormones Methyl Jasmonate or Salicylate Had No Effect on the Growth of *Rhizoctonia solani* Colony at Concentrations Used for Priming Tomato Plants

In order to check if the phytohormones methyl jasmonate or salicylate had any effect on the growth of *R. solani*, the pathogen was grown on PDA that was supplemented with either MeJA or SA at five different concentrations, viz., 0.05, 0.08, 0.1, 0.5, and 1 mM. Mycelial growth was recorded for 3 consecutive days (Supplementary Figures 5, 6). Results show that MeJA had no effect on the growth of mycelia up to the concentration of0.1mM (Supplementary Figure 7A), which was above the concentration of0.08 mM used in the priming experiments. For SA, there was no effect on the mycelial growth at a concentration as high as0.5 mM, which was above the concentration of0.08 mM used for priming of tomato plants (Supplementary Figure 7B).

## Discussion

This present study of host-pathogen interaction between *Rhizoctonia solani* and phytohormone-primed tomato plants revealed some unique findings. There is a general perception that SA-mediated defense is induced against biotrophic pathogens and JA-mediated defense functions against necrotrophic pathogens (Glazebrook, 2005). Based on this perception, in previous studies on necrotrophic pathogens, the hosts have been primed with only JA (Oliveira et al., 2015; Yu et al., 2018). In this study, we included SA priming and compared its effect with that imparted by MeJA priming. This new strategy gave us some unexpected insights. Observations were made from the point of altered behavior of the pathogen and the altered defense response of the host in response to the pathogen in regards to phytohormone priming of the host.

In our earlier reports, we had established that fungi can and do differentiate between a tolerant and susceptible host from a distance and behave differently when placed in the vicinity of the host (Chowdhury et al., 2014; Basu et al., 2016, Chowdhury et al., 2017a). We had showed that fungi also behave differently on the surface and within the host tissues of tolerant and susceptible hosts. In these earlier reports, the fungal pathogens *Macrophomina phaseolina* and *R. solani* showed nearly double the growth of hyphae toward a susceptible variety compared to a tolerant variety of sesame and rice, respectively, and during establishment phase, showed clear avoidance in coming into close contact with the tolerant host surface. In the present study, we wanted to see for the first time if a fungus could detect phytohormone primed versus unprimed control hosts from a distance and behave differently in their vicinity, on the surface, and within the host tissue. In fact, *R. solani* did show significant differences in its behavior, exhibiting a clear preference for the unprimed plants by growing quickly and directly toward the leaves, making almost perpendicular contacts. This indicates that the fungus can detect an unprimed host as a more viable option and focus its growth toward that direction. On the surface of control leaves, the hyphal network made intimate contact with the leaf surface and interacted with the trichomes of control plants while avoiding intimate contact with the primed leaf surface. All these observations are likely due to the ability of the fungus to detect favorable exudates from the unprimed host from a distance or to detect some kind of inhibitor from the primed hosts or both. Similarly, the behavior of sclerotia showed a preference for the control leaves over the primed leaves, showing earlier germination on control leaves, while on the primed leaves, the sclerotia showed a reluctance to germinate. More surprisingly, the sclerotia showed most delayed germination on SA pre-treated leaves, which was less than MeJA pretreatment.

In our earlier report on rice, we have shown that during *R. solani* infection, the formation of infection cushions and lobate appressoria is a direct measure of disease progression (Basu et al., 2016). Disease severity and tissue necrosis have been positively correlated with the number of these two structures (Nikraftar et al., 2013; Basu et al., 2016). In the present study, in the SA-treated plants, formation of infection cushions, lobate appressoria, and hyphal progression was visibly lower than MeJA pre-treated plants. Overall, the fungus showed clear avoidance in all three locations, i.e., in the vicinity of host, on host surface, and in the post-invasion stages in the phytohormone primed plants, especially when primed with SA. We had established earlier that this type of difference in hyphal preference toward host is directly attributed to the tolerance level of the host (Chowdhury et al., 2014, 2017a; Ray et al., 2015; Basu et al., 2016).

To find a molecular basis of this difference, expression of the fungal *GAS1* gene, which is directly linked with appressorial penetration (Ghosh et al., 2018), was assayed over a time course under different priming regime. Corroborating the microscopy data, the expression level of *GAS1* was least in SA pre-treated plants followed by MeJA and control. Considering that *R. solani* is a necrotrophic fungus, these results go against the general perception that SA-mediated defense works against biotrophic and hemibiotrophic pathogens (Chowdhury et al., 2017a; Qi et al., 2018, Djavaheri et al., 2019), whereas JA-mediated defense is directed against necrotrophs (Bürger and Chory, 2019; Brenya et al., 2020).

While callose provides a physical barrier against pathogen invasion (Chowdhury et al., 2017a; Wang et al., 2019), several other biochemical constituents like polyphenols and flavonoids also accumulate during infection (Gillmeister et al., 2019). Callose was seen to accumulate more significantly after infection in the SA pretreated plants compared to control and MeJA-primed plants. Interestingly, we have observed that only half of the stoma, i.e., one guard cell fluoresced first then, subsequently, both of the guard cells fluoresced in the SA-primed plants in response to *R. solani* infection. We could not find any earlier research in this regard. Another interesting observation was that in the SA-primed plants, significantly higher cellular phenol deposition was observed at earlier stages of infection (2dpi) while in the later stages (3dpi), phenol was significantly higher in MeJA-primed plants. Quantitative assay of total phenolics and flavonoids also followed the same pattern. Regulation at the gene expression level was analyzed by observing the expression profiles of the flavonoid synthesizing genes like *CHS1* and *CHS2* over a time course during infection. These two genes have been linked to the induced defense in hosts against hemibiotrophic pathogens under different conditions, including SA priming of the host (Campos et al., 2003; Król et al., 2015). While phytohormone priming limited the extent of degradation of chlorophyll-a throughout the course of pathogenesis, Chl-a was least degraded in SA-treated plants especially in the early time points of infection compared to MeJA. All these observations, taken together, indicate that activated defense through the secondary metabolites was directed by the salicylate pathway in the early phase of pathogenesis which was taken over at later stages of infection by the jasmonate signaling pathway.

The appropriate balance between accumulation of ROS and removal of ROS is an important target for the pathogen and the host for the sake of their own survival (Kou et al., 2019). Although there are numerous studies in this area of research, the molecular mechanism involved in H_2_O_2_ mediated regulation of plant systemic defense responses against infection is not clear (Zhang et al., 2021). In our experiments, H_2_O_2_ accumulation was maximum in the control plants followed by MeJA-primed and least in the SA-primed plants. Although this may apparently seem to be unexpected, the less H_2_O_2_ in the primed plants can be attributed to three factors. The lower amount of H_2_O_2_ was due to significantly less infection in these plants and less disease progression at these respective time points. Also, according to earlier reports, there is a direct correlation between H_2_O_2_ content and degradation of chlorophyll (Shetty et al., 2007). Moreover, the pathogen itself is often the source of the excess H_2_O_2_ (Shetty et al., 2007; Kant et al., 2019). All these factors can explain the lower H_2_O_2_ in the SA-primed plants since due to less disease progression, there was less chlorophyll degradation and less pathogen load, and hence, less sources of H_2_O_2_ in these plants.

Plant fitness is largely dependent on the perfect trading-off in recognizing the nature of the encountered pathogen and the type of induced defense activated (Vos et al., 2015). *R. solani* is traditionally designated as a necrotrophic pathogen. Therefore, the major unexpected observation was that SA pre-treated plants showed better defense response than MeJA-treated ones and that jasmonate-mediated defense set in after the initial SA signaling phase. To corroborate these findings, the expression of a total of twelve marker genes functioning in the SA and JA signaling pathways were monitored over a time course. In our experiments, priming with exogenous application of SA and subsequent infection with the pathogen activated jasmonate signaling through the *COI-JAZ* component. Liu et al. (2016) showed that an increased level of SA near the infection site activates JA signaling by release of *JAZ* repression, along with *de novo* JA synthesis during infection by the hemibiotroph *Pseudominas syringe* on *Arabidopsis*. Even though our pathosystem included a, thus far, designated necrotrophic pathogen, we observed that *COI* was upregulated at earlier time points post-infection in SA-primed plants than MeJA-primed plants. Another one of our observations was that in the SA-primed plants, there was less expression of repressor than MeJA-primed plants. This was unexpected since *R. solani* is considered as a necrotroph and MeJA-mediated defense is employed by plants against necrotrophs. Our data matches with hemibiotroph pathosystem where Liu et al. (2016) showed that with the production of endogenous SA around the wound site, there was concomitant degradation of the repressor JAZ during the hemibiotroph infection.

Corroborating this earlier report on *de novo* synthesis of JA by localized production of SA by Liu et al. (2016), our data further show activation of *AOS*, the JA synthesis gene, in SA-primed plants at 24 hpi. *AOS* level can be elevated by either SA itself (Laudert and Weiler, 1998) or by a SA analog (Halim et al., 2009) with the latter conferring resistance against hemibiotrophic pathogen. Based on these results, it can be said that in the present study, induced JA signaling in the later phase with concomitant upregulation of JA synthesizing gene *AOS* along with the JA content in SA-primed background in the later phase of infection likely contributed to resistance against *R. solani*.

Systemin receptor 160 (SR160) is a cell-surface receptor of systemin, the polypeptide that is produced by injured host tissue, the interaction of which leads to defense response (Wang et al., 2018). *SR160* is considered as an early defense gene that is induced within a few hours in response to either wounding or systemin and initiates JA dependent tolerance (Kandoth et al., 2007). In our study, in the control plants, *SR160* was significantly upregulated within 4 hpi. Contrastingly, exogenous application of JA and SA was able to delay the upregulation of *SR160* gene till 24 hpi. Since the phytohormone pre-treatment delayed the onset of the disease, the release of systemin from damaged tissues and its receptor gene was automatically delayed. Most importantly, SA pre-treatment showed significantly less SR160 transcript levels even at 24 hpi compared to MeJA-primed plants, corroborating the fact that SA priming contributed to less tissue damage and greater tolerance to the pathogen than MeJA priming.

It has been well established that *COI*-mediated release of transcriptional inhibition by JAZ repressors leads to JA defense cascades (Guo et al., 2018). In the present study, the defense route involved in MeJA-treated plants against *R. solani* pathogenesis was found to be *COI1/JAZ*-mediated induction of *PINII*. The extremely high expression of *PINII* in the first 8 hpi was therefore due to the direct effect of MeJA priming. SA can suppress the octadecanoid pathway and accumulation of *PIN-II* in JA-treated tomato seedlings (Zhang et al., 2021). This can explain our observation that there was a decline of *PINII* in SA-primed plants up to 8 hpi (i.e., 32 hours post-priming) and then gradually rising due to infection.

In plants, SA biosynthesis is proposed to take place through two pathways. The bulk of SA is synthesized from isochorismate through isochorismate synthase (*ICS*). SA is also synthesized from cinnamate produced by the activity of phenylalanine ammonia lyase (*PAL*) (Chen et al., 2009). In our experiments, SA priming was inducive to *ICS* upregulation which increased significantly at 1 day post-inoculation. Unlike SA, MeJA pretreatment was not found to be inductive for SA synthesis *via* the isochorismate route, as reflected by the complete absence of the *ICS* transcripts in MeJA-primed plants. Surprisingly, MeJA priming induced the SA biosynthesis-related enzyme gene, *PAL5*, later in the infection period, even more so than SA priming. Chen et al. (2017) observed increased PAL5 content after MeJA priming in response to symbiont infection. Our pathogen, in spite of being a necrotroph, elicited similar host response. Ferrari et al. (2003) observed that resistance to *Botrytis cineria* was mediated through the SA pathway and that the biosynthesis was routed through *PAL* rather than *ICS.* Although originally designated as a necrotroph, *B. cineria* was later proposed by van Kan et al. (2014) to be an endophyte and not a true necrotroph.

We included the gene for *BSMT* (BA/SA carboxyl methyltransferase 1) enzyme which leads to the formation of volatile methyl salicylate (Li et al., 2019). In this inactive form, methyl salicylate can be stored in cells for future use (Maruri-López et al., 2019). SA priming not only induced the SA biosynthesis gene *ICS* as there was less expression of the SA metabolizing gene *BSMT* in the SA-primed plants. This suggests a strong role for SA in tolerance to *R. solani*. Furthermore, MeJA priming enhanced SA metabolism through *BSMT* gene expression in the late phases, showing a shift toward the JA-mediated defense pathway in the later phases. In contrast, in control sets, *BSMT* peaked in the early phases. Hence, in the control plants, there is less SA available in the early phases of infection and this likely contributed to less tolerance. Again, these observations are not consistent with typical defense signaling against a necrotroph. This is rather consistent with earlier report that *COR*-mediated enhanced *BSMT* expression increased host susceptibility to the hemibiotroph *Pseudomonas syringae* infection by converting free SA to inactive MeSA (Zheng et al., 2012).

Another crucial component of in SA-mediated defense is the pathogenesis-related (PR) protein. Resistance is mediated through the activation of *PR* genes and subsequent activation of downstream SA signaling pathway (Ali et al., 2018). In this study, induced PR1a expression in the phytohormone treated plants, especially in SA-primed plants, inhibited growth and establishment of the pathogen. PR protein genes are known to be upregulated in biotrophic and hemibiotrophic pathogenic interactions (Chowdhury et al., 2017a; Ali et al., 2018, Boccardo et al., 2019).

Earlier studies show that exogenous application of SA increases expression of PR (Chen et al., 2017) and that H_2_O_2_ does not function downstream of SA in the induction of PR protein expression (Bi et al., 1995; Zhang et al., 2021). Consistent with earlier studies, our results show that even though there was less H_2_O_2_ in the SA-primed plants compared to control plants (due to less infection), the *PR1* gene expression was higher than that of control at all time points in the SA-primed plants.

Moreover, *ICS*, which is the major SA biosynthesis gene, is known to induce SA accumulation and activation of SA signaling network through EDS1/PAD4 after pathogen infection (Makandar et al., 2015; Shine et al., 2016). This corroborates our observation that there is a sharp increase in SA content within the first day after infection and that along with *ICS*, *PAD4* is also upregulated in the SA-primed plants after infection. Collectively, these data indicate an important role of salicylate in disease resistance against *R. solani* in tomato, especially in the earlier phases of pathogenesis.

In the field of phytohormone defense, there is a general idea of segregation of SA and JA function with respect to trophic nature of the invading microorganism where SA is the major phytohormone involved against biotrophic pathogens (Quentin et al., 2016), while JA functions against necrotrophic pathogens (Oliveira et al., 2015; Yu et al., 2018). In the present study, the response of the pathogen toward MeJA and SA pre-treated plants and subsequent defense response in the host point at the coordinated activation of SA followed by JA defense signaling with SA priming enhancing the disease tolerance better than JA priming.

In our earlier report (Chowdhury et al., 2017a), the interplay between the two hormonal defense pathways had been observed and established for the first time in a so-called necrotroph, *Macrophomina phaseolina*, which was found to have a short biotrophic phase before the necrotrophic phase. We had established that the switch of this pathogen from biotrophic to necrotrophic phase was accompanied by a switch in the host defense strategy from SA-mediated signaling in the early phases to JA-mediated signaling in the later phases of infection (Chowdhury et al., 2017a). This switch was more prompt in the tolerant variety of hosts than susceptible hosts which contributed to better disease tolerance (Chowdhury et al., 2017a). Our report was corroborated by another group with transcriptome analysis using the same pathosystem (Yan et al., 2021). Later, in a similar report by Schroeder et al., 2019, with *Arabidopsis* as host, the pathogen evoked mainly SA-mediated defense response in early phases which shifted to JA/ET alone in the later phases. Our finding that *CHS1* and *CHS2* has been increased in SA-primed plants in response to infection is similar to the observations made by Campos et al., 2003 on the hemibiotrophic pathogen *Colletotrichum* sp. Also, Ding et al. (2011) observed a biphasic expression of SA followed by JA-related genes was observed in the first 24 h during infection by the hemibiotroph, *F. graminearum*, in wheat. Finally, van den Berg et al. (2018) observed a similar two-phased expression of SA followed by JA-related genes in the first day of infection by the hemibiotroph *Phytophthora* on avocado. Hence, the present scenario of biphasic SA-JA defense cascades during *R. solani* interaction in primed plants, and with SA priming imparting maximum tolerance during disease development in tomato, this indicates a possible hemibiotrophic pathosystem that needs to be investigated further.

## Data Availability Statement

The original contributions presented in the study are included in the article/Supplementary Material, further inquiries can be directed to the corresponding author/s.

## Author Contributions

SK designed the project, drafted the experiments, and procured the funding. PK, SB, AS, and SL performed the experiments and analyzed data. SK, PK, SB, and AS wrote the manuscript. CD, MM, MD, ND, AD, and MT helped with the experiments and writing of the manuscript. All authors contributed to the article and approved the submitted version.

## Conflict of Interest

The authors declare that the research was conducted in the absence of any commercial or financial relationships that could be construed as a potential conflict of interest.

## Publisher’s Note

All claims expressed in this article are solely those of the authors and do not necessarily represent those of their affiliated organizations, or those of the publisher, the editors and the reviewers. Any product that may be evaluated in this article, or claim that may be made by its manufacturer, is not guaranteed or endorsed by the publisher.

## Supplementary Material

Supplementary Figure 1 RT-qPCR analysis of the expression of Jasmonate signaling marker genes in primed and control plants post infection with *R. solani*. Graphs of Quantitative real-time RT-PCR analysis of genes at 24, 48, and 72 h post-inoculation: **(A)** *CORONATINE INSENSITIVE 1* (*COI1*), **(B)** Jasmonate ZIM (zinc-finger inflorescence meristem) domain (*JAZ2*), **(C)** Allene oxide synthase (*AOS*), **(D)** Systemin receptor SR160 (*SR160*), **(E)** Proteinase inhibitor II (*PIN II*). Bars represent standard error (SE) of the mean (*n* = 3). Different letters indicate significant differences among treatments at *p* < 0.05, according to Duncan’s multiple range test.

Supplementary Figure 2 RT-qPCR analysis of the expression of Salicylate signaling marker genes in primed and control plants post-infection with *R. solani*. Graphs of Quantitative real-time RT-PCR analysis of genes at 24, 48, and 72 h post-inoculation: **(A)** Isochorismate synthase (*ICS*), **(B)** Phenylalanine ammonia lyase 5 (*PAL5*), **(C)** Salicylate/benzoate carboxyl methyltransferase (*BSMT*), **(D)** Pathogenesis-related protein-1a (*PR1a*), **(E)** Phytoalexin-deficient 4 *(PAD4*). Bars represent standard error (SE) of the mean (*n* = 3). Different letters indicate significant differences among treatments at *p* < 0.05, according to Duncan’s multiple range test.

Supplementary Figure 3 Quantification of salicylic acid (SA) and jasmonic acid (JA) content in control and primed plants post-infection with *R.solani* over a time course: **(A)** SA content, **(B)** JA content. Bars represent standard error (SE) of the mean (*n* = 3). Different letters indicate significant differences among treatments at *p* < 0.05, according to Duncan’s multiple range test.

Supplementary Figure 4 Quantification of SA and JA content in control and primed plants over a time course after priming. **(A)** SA content, **(B)** JA content. Bars represent standard error (SE) of the mean (*n* = 3). Different letters indicate significant differences among treatments at *p* < 0.05, according to Duncan’s multiple range test.

Supplementary Figure 5 Culture of *Rhizoctonia solani* on potato dextrose agar (PDA) media supplemented with MeJA in increasing concentrations to assay the effect of MeJA on the growth of the fungus. Bar = 2cm.

Supplementary Figure 6 Culture of *Rhizoctonia solani* on PDA media supplemented with SA in increasing concentrations to assay the effect of SA on the growth of the fungus. Bar = 2 cm.

Supplementary Figure 7 Graph showing the measurement of diameter of *R. solani* colony on PDA supplemented with phytohormones in increasing concentrations **(A)** MeJA, **(B)** SA.
