# Supplementary figures and images for: Phytohormone Priming of Tomato Plants Evoke Differential Behavior in Rhizoctonia solani During Infection, With Salicylate Priming Imparting Greater Tolerance Than Jasmonate
Source: Front Plant Sci. 2022 Jan 10;12:766095. doi: 10.3389/fpls.2021.766095 (PMC8784698; doi:10.3389/fpls.2021.766095)

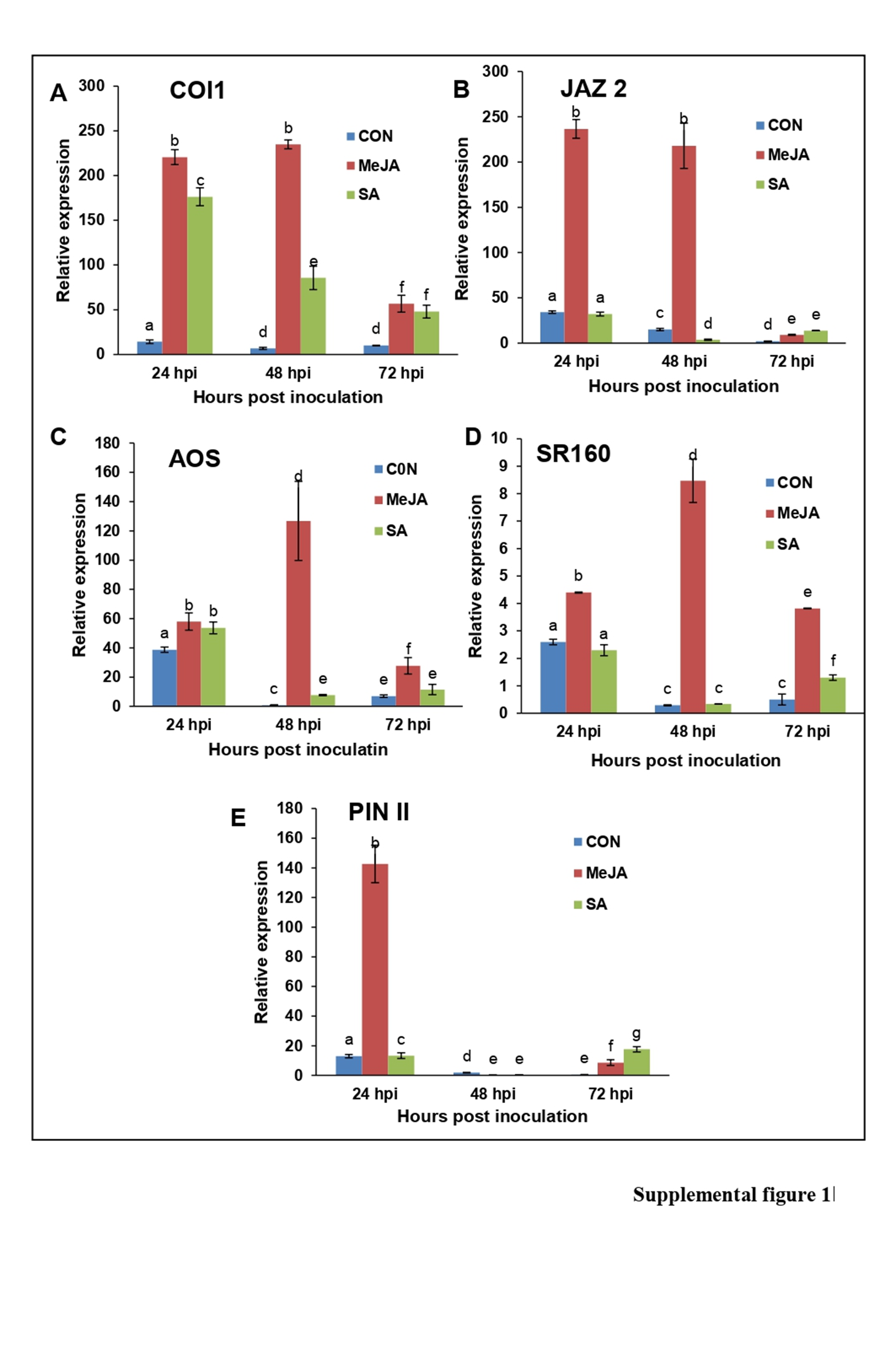

Supplement: Supplementary Figure 1 — RT-qPCR analysis of the expression of Jasmonate signaling marker genes in primed and control plants post infection with R. solani. Graphs of Quantitative real-time RT-PCR analysis of genes at 24, 48, and 72 h post-inoculation: (A) CORONATINE INSENSITIVE 1 (COI1), (B) Jasmonate ZIM (zinc-finger inflorescence meristem) domain (JAZ2), (C) Allene oxide synthase (AOS), (D) Systemin receptor SR160 (SR160), (E) Proteinase inhibitor II (PIN II). Bars represent standard error (SE) of the mean (n = 3). Different letters indicate significant differences among treatments at p < 0.05, according to Duncan’s multiple range test. [file Image_1.TIFF]

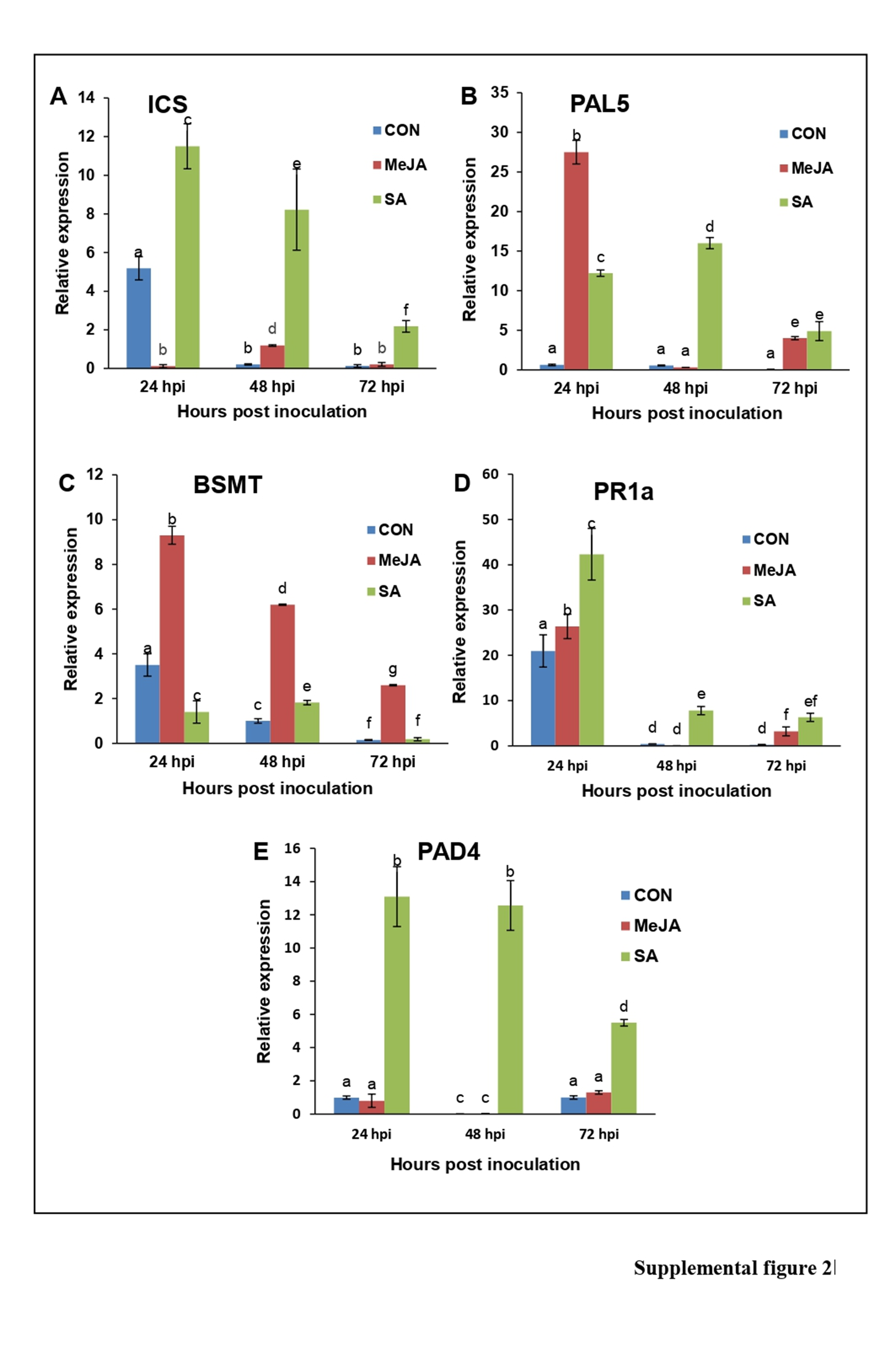

Supplement: Supplementary Figure 2 — RT-qPCR analysis of the expression of Salicylate signaling marker genes in primed and control plants post-infection with R. solani. Graphs of Quantitative real-time RT-PCR analysis of genes at 24, 48, and 72 h post-inoculation: (A) Isochorismate synthase (ICS), (B) Phenylalanine ammonia lyase 5 (PAL5), (C) Salicylate/benzoate carboxyl methyltransferase (BSMT), (D) Pathogenesis-related protein-1a (PR1a), (E) Phytoalexin-deficient 4 (PAD4). Bars represent standard error (SE) of the mean (n = 3). Different letters indicate significant differences among treatments at p < 0.05, according to Duncan’s multiple range test. [file Image_2.TIFF]

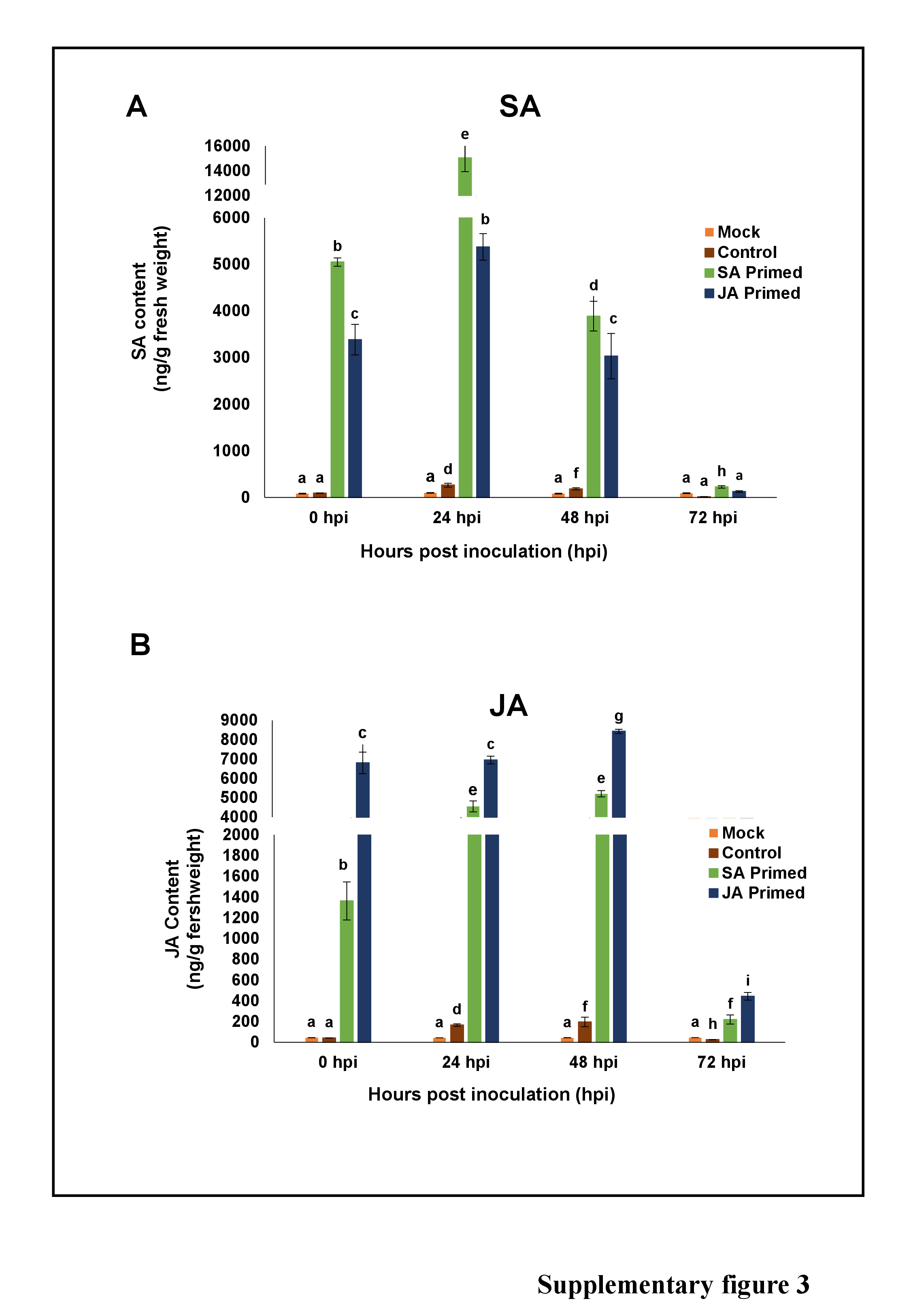

Supplement: Supplementary Figure 3 — Quantification of salicylic acid (SA) and jasmonic acid (JA) content in control and primed plants post-infection with R.solani over a time course: (A) SA content, (B) JA content. Bars represent standard error (SE) of the mean (n = 3). Different letters indicate significant differences among treatments at p < 0.05, according to Duncan’s multiple range test. [file Image_3.TIFF]

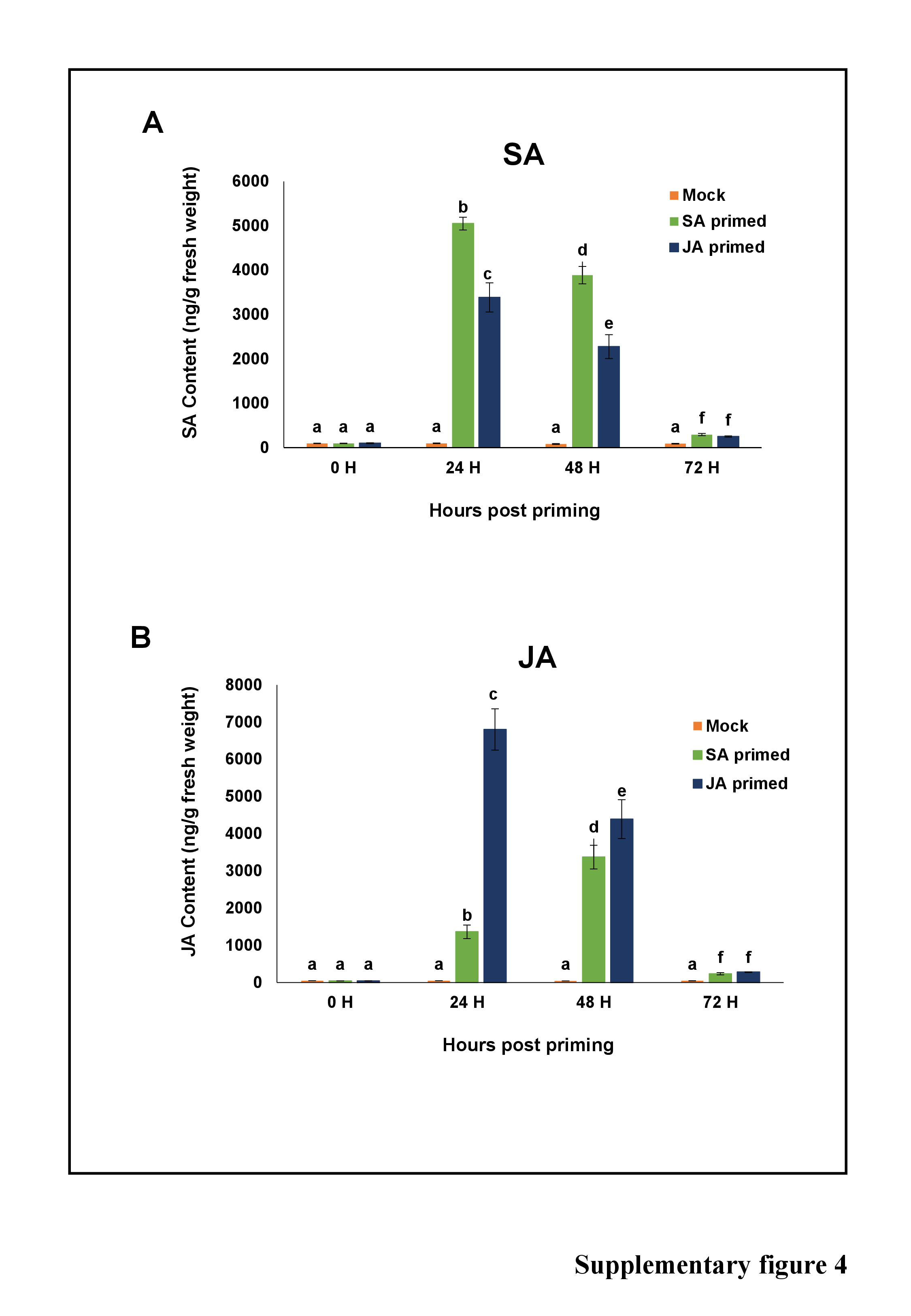

Supplement: Supplementary Figure 4 — Quantification of SA and JA content in control and primed plants over a time course after priming. (A) SA content, (B) JA content. Bars represent standard error (SE) of the mean (n = 3). Different letters indicate significant differences among treatments at p < 0.05, according to Duncan’s multiple range test. [file Image_4.TIFF]

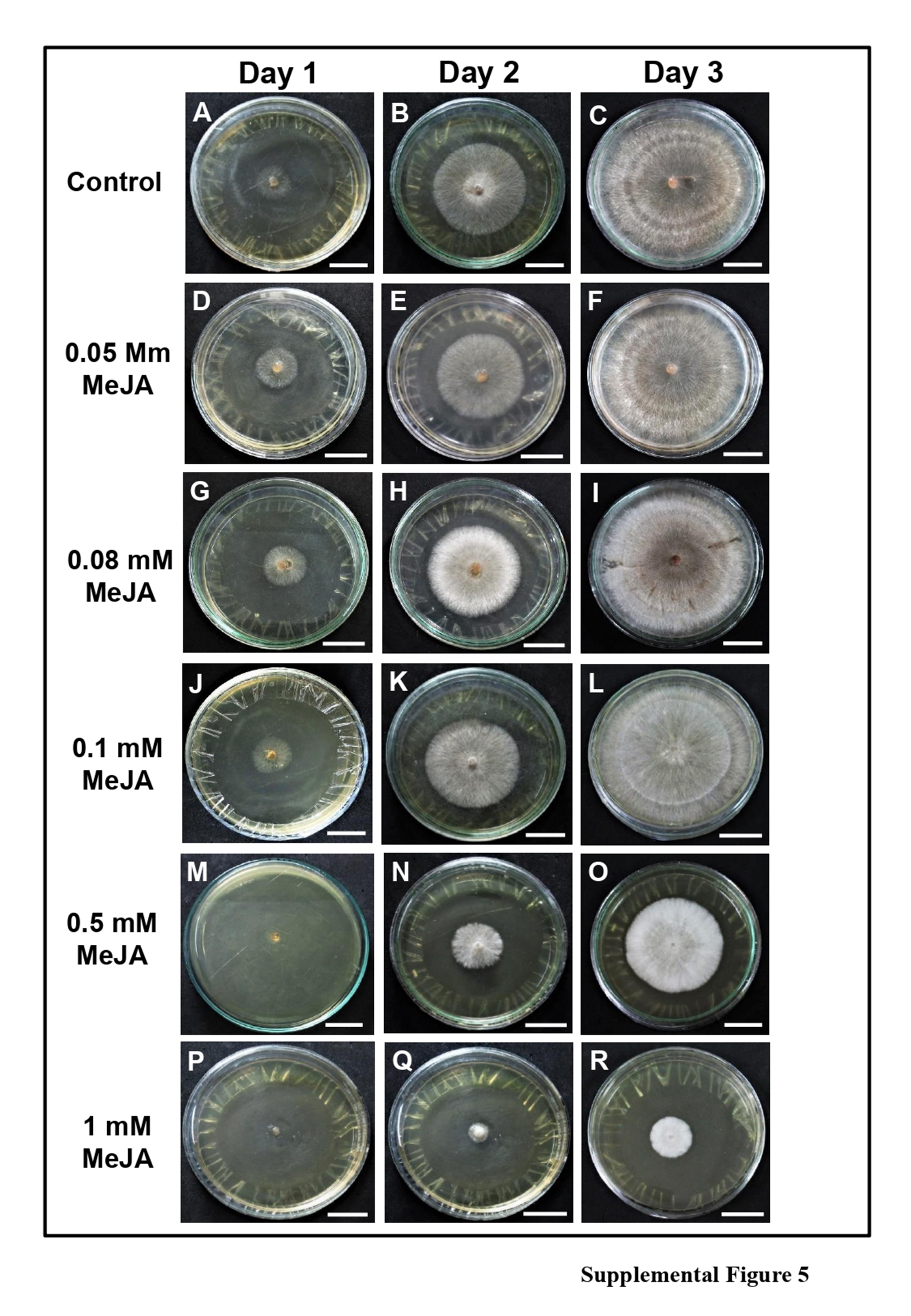

Supplement: Supplementary Figure 5 — Culture of Rhizoctonia solani on potato dextrose agar (PDA) media supplemented with MeJA in increasing concentrations to assay the effect of MeJA on the growth of the fungus. Bar = 2cm. [file Image_5.TIFF]

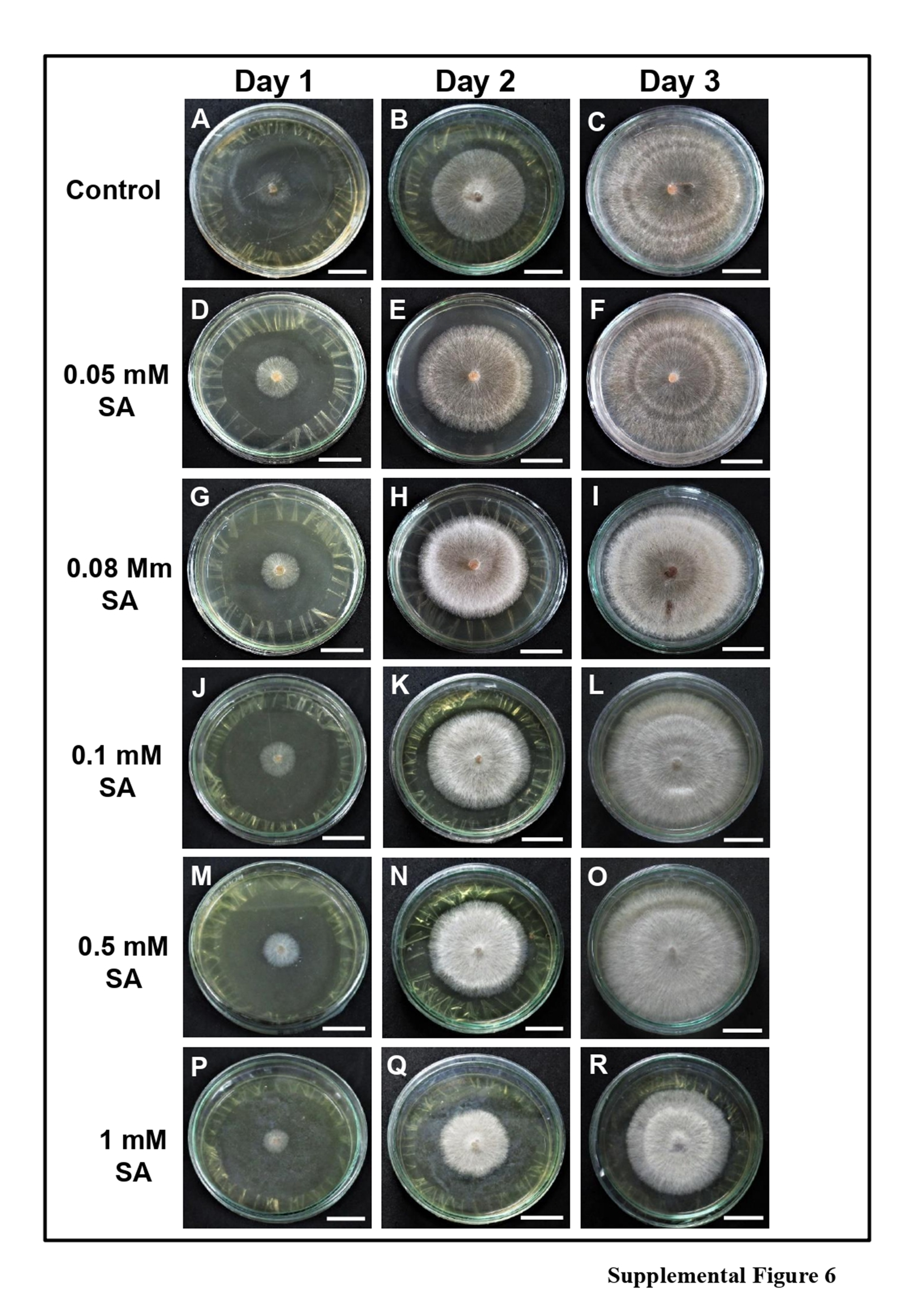

Supplement: Supplementary Figure 6 — Culture of Rhizoctonia solani on PDA media supplemented with SA in increasing concentrations to assay the effect of SA on the growth of the fungus. Bar = 2 cm. [file Image_6.TIFF]

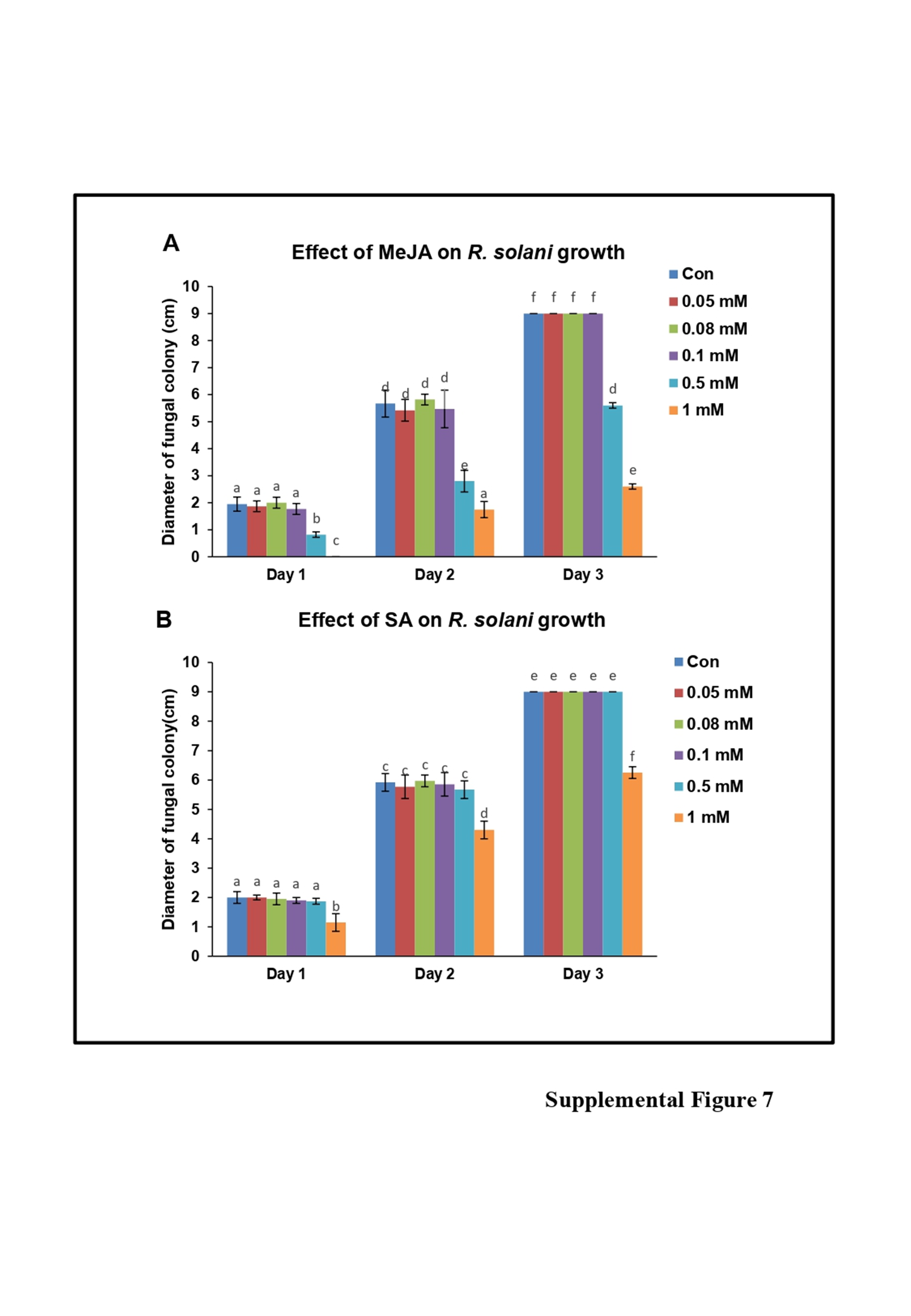

Supplement: Supplementary Figure 7 — Graph showing the measurement of diameter of R. solani colony on PDA supplemented with phytohormones in increasing concentrations (A) MeJA, (B) SA. [file Image_7.TIFF]
